# Patients’ Perceptions Toward Human–Artificial Intelligence Interaction in Health Care: Experimental Study

**DOI:** 10.2196/25856

**Published:** 2021-11-25

**Authors:** Pouyan Esmaeilzadeh, Tala Mirzaei, Spurthy Dharanikota

**Affiliations:** 1 Department of Information Systems and Business Analytics College of Business Florida International University Miami, FL United States

**Keywords:** AI clinical applications, collective intelligence, in-person examinations, perceived benefits, perceived risks

## Abstract

**Background:**

It is believed that artificial intelligence (AI) will be an integral part of health care services in the near future and will be incorporated into several aspects of clinical care such as prognosis, diagnostics, and care planning. Thus, many technology companies have invested in producing AI clinical applications. Patients are one of the most important beneficiaries who potentially interact with these technologies and applications; thus, patients’ perceptions may affect the widespread use of clinical AI. Patients should be ensured that AI clinical applications will not harm them, and that they will instead benefit from using AI technology for health care purposes. Although human-AI interaction can enhance health care outcomes, possible dimensions of concerns and risks should be addressed before its integration with routine clinical care.

**Objective:**

The main objective of this study was to examine how potential users (patients) perceive the benefits, risks, and use of AI clinical applications for their health care purposes and how their perceptions may be different if faced with three health care service encounter scenarios.

**Methods:**

We designed a 2×3 experiment that crossed a type of health condition (ie, acute or chronic) with three different types of clinical encounters between patients and physicians (ie, AI clinical applications as substituting technology, AI clinical applications as augmenting technology, and no AI as a traditional in-person visit). We used an online survey to collect data from 634 individuals in the United States.

**Results:**

The interactions between the types of health care service encounters and health conditions significantly influenced individuals’ perceptions of privacy concerns, trust issues, communication barriers, concerns about transparency in regulatory standards, liability risks, benefits, and intention to use across the six scenarios. We found no significant differences among scenarios regarding perceptions of performance risk and social biases.

**Conclusions:**

The results imply that incompatibility with instrumental, technical, ethical, or regulatory values can be a reason for rejecting AI applications in health care. Thus, there are still various risks associated with implementing AI applications in diagnostics and treatment recommendations for patients with both acute and chronic illnesses. The concerns are also evident if the AI applications are used as a recommendation system under physician experience, wisdom, and control. Prior to the widespread rollout of AI, more studies are needed to identify the challenges that may raise concerns for implementing and using AI applications. This study could provide researchers and managers with critical insights into the determinants of individuals’ intention to use AI clinical applications. Regulatory agencies should establish normative standards and evaluation guidelines for implementing AI in health care in cooperation with health care institutions. Regular audits and ongoing monitoring and reporting systems can be used to continuously evaluate the safety, quality, transparency, and ethical factors of AI clinical applications.

## Introduction

### Artificial Intelligence

Artificial intelligence (AI) generally refers to a computerized system (hardware or software) that can perform physical tasks and cognitive functions, solve various problems, or make decisions without explicit human instructions [1]. A range of techniques and applications are under the broad umbrella of AI, such as genetic algorithms, neural networks, machine learning, and pattern recognition [2]. AI is considered a frontline service technology (FST) in the literature [3]. FST infusion in various industries has emerged as a topic of interest in the past decade (eg, [4-8]). Gursoy et al [9] classified FST infusion under three main categories: (1) no FST, which refers to a technology-free encounter between consumers and frontline service providers; (2) augmenting FST, which refers to the technology as a human augmentation tool; and (3) substituting FST, which refers to the technology as a human substitution force. In augmenting FST, the technology can help to enhance human thinking, analysis, and behavior, and boost the ability to interact with other human actors, whereas in substituting FST, the technology substitutes a human actor and takes away the active role of humans in the service encounter. AI as an FST can augment or replace human tasks and activities within a wide range of industrial, intellectual, and social applications with potential impacts on productivity and performance. As nonhuman intelligence is programmed to complete specific tasks, AI can overcome some of humans’ computationally intensive and intellectual limitations [10]. For example, AI could be a computer application that uses sophisticated algorithms to solve a business problem for managers. AI applications generate personalized recommendations to customers based on analysis of a huge data set. Thus, it is believed that AI could perform tasks better than the best humans and experts in any field [2].

AI technology, including algorithmic machine learning and autonomous decision-making, creates new opportunities for continued innovation in different industries, including finance, health care, manufacturing, retail, supply chain, logistics, and utilities [11]. Promoting AI applications has become one of the focal points of many companies’ strategies [12]. The notable changes made by AI have inspired recent studies to examine the impacts and consequences of the technology and investigate AI’s performance implications. However, this objective requires an in-depth understanding of the factors affecting the acceptance of AI applications by potential users in different manufacturing and service fields.

### AI in Health Care

Previous studies highlight the importance of AI in health care, especially in medical informatics [13]. AI can improve patient care, diagnosis, and interpretation of medical data [14]. Houssami et al [15] showed that AI applications used for breast cancer screening reduced human detection errors; however, some of the interrelated ethical and societal trust factors, as well as reliance on AI, are yet to be developed. Prior research has shown that AI clinical applications exhibit the same or sometimes even better performance than their human counterparts or specialists in detecting Alzheimer disease using natural language processing techniques [16], and for detecting skin cancer [17] and heart arrhythmia [18] using deep neural networks. AI applications for health care recommendations may differ from those in other sectors, mainly because of the highly sensitive nature of health information and high levels of consumer vulnerability to possible medical errors.

In the context of AI in health care, there can be three possible patient encounters for care delivery. First, the patient can follow the traditional health care delivery model and visit a physician in person. This option is the most prevalent health care delivery process that focuses on the physician-human interaction and can be referred to as a “human-human interaction” or “traditional in-person visit.” Second, a patient can choose a collaborative intelligence [19] scenario where the physicians collaborate with an AI application to arrive at conclusions and make medical decisions. In other words, the physician’s thinking, analysis, and performance are augmented by using AI applications to interact with patients, but the ultimate responsibility of patient care and recommending treatment options and care planning still rest with the physician. Third, AI clinical applications substitute the physician, and a patient only encounters the AI clinical application unaided by human intelligence.

### Acceptance of AI in Clinical Applications

In April 2018, the Food and Drug Administration authorized the first AI application to diagnose diabetic retinopathy without a physician’s help in the United States [20]. An increasing number of health care service companies have invested in AI applications in mobile health devices or health apps to improve patient safety, increase practice quality, enhance patient care management, and decrease health care costs. However, previous studies suggest that not all individuals are willing to accept AI clinical applications [20]. Successful implementation of AI applications requires a careful examination of users’ attitudes and perceptions about AI [21]. Thus, investing in AI applications without recognizing potential users’ beliefs and willingness to use them may waste resources and even result in customer loss. This is especially true in the health care sector, where patient engagement is considered one of the most critical determinants of health care quality. If individuals do not view interacting with AI clinical applications as useful, they may demand interactions with physicians, and in turn, the AI applications may remain unused. Therefore, understanding the decision drivers and barriers that lead to acceptance or refusal of AI clinical applications in health care delivery is fundamental for health care providers and hospitals that plan to introduce or increase AI presence during health care delivery.

Several studies have investigated the attitude of specific sample populations toward AI in health care. For example, Dos Santos and colleagues [22] evaluated medical students’ attitudes toward AI and found that the majority agreed that AI could improve medicine as a whole. In a similar study involving medical students in Canada [23], the majority of the sample (67%) agreed that AI would reduce the demand for medicine and 47% were anxious about physicians’ future in association with AI. In a survey conducted among members of the European Society of Radiology [24], more than half of the respondents strongly expressed that they were not ready to accept AI-only generated reports. Similarly, in a study investigating perceptions toward health care robots [25], residents in a retirement village exhibited a more positive attitude toward the robot compared to the responses of their staff and relatives. Recently, Patel and colleagues [26] compared the diagnosis results of pneumonia on chest radiographs among human experts alone, collaborative intelligence, and two state-of-the-art AI deep learning models. They demonstrated that both the collaborative and AI models have superior performance when compared with human experts alone. They also found that the combination of collaborative and AI models outperforms each of these methods alone.

### Research Gaps

Based on previous studies, health care professionals still express fundamental concerns about implementing AI clinical applications in care services [27-30]. These concerns and risks also directly affect patients’ perceptions (as potential users and beneficiaries) and make them withdraw from using AI clinical applications [20]. The majority of studies investigating attitudes toward AI involve national samples [31], medical students [22], radiology experts [23], and physicians [27], and we did not find a study that examined the perceptions of patients with different health conditions (chronic and acute diseases) toward AI clinical applications. Exploring the perspectives of individuals with different diseases can allow researchers to effectively recognize the source of risks and concerns associated with AI clinical applications. Since the risks may vary by type of illness, this perspective can help health care providers better understand how to address the concerns of various patients.

Thus, researchers need to understand the current challenges related to AI technologies more efficiently and analyze the urgent needs of health systems to design AI applications to address them. Even with physicians and other health care stakeholders accepting and assimilating AI to varying degrees, it is crucial to understand patients’ perspectives toward these different scenarios. Nevertheless, little is known about the risk beliefs associated with using AI clinical applications for diagnosis and treatments from the general public’s perspective. This stream of literature encouraged us to examine people’s perceptions and attitudes toward different types of health care delivery processes in this study.

Currently, the issues related to AI clinical applications in health care are still within the realm of research. However, it is widely believed that these systems will fundamentally change medical practice in the near future [32]. Historically, the medical sector does not integrate technology as quickly as other industries [33]. Moreover, integrating AI into the current medical workflow could be very challenging without the involvement, cooperation, and endorsement of stakeholders (such as health care professionals and patients) and a robust legislative and regulatory framework. The main objective of this study was to examine how potential users perceive the benefits, risks, and use of AI clinical applications for their health care purposes, and how their perceptions may be different if faced with the three health care service encounter scenarios. The benefit perceptions and risk beliefs of prospective users may affect their future adoption of AI applications. Patients may not decide what tools health care professionals should use in their practice, but they can definitely highlight possible concerns, challenges, and barriers that may refrain them from supporting and using the tools implemented and promoted by clinicians.

### Literature Review

#### Overview

In this section, consistent with the research objectives, three topics are explained. First, the type of illness and the reactions of people with different illnesses (acute or chronic) are described. Second, possible risks and concerns associated with the use of AI clinical applications are highlighted. Third, potential benefits that users may perceive from using AI in health care are illuminated. The interrelationships among these three topics can provide further research background on how people with different health conditions may react to AI applications used for health care purposes to place our research objectives and experimental design in context.

#### Type of Illness (Acute or Chronic)

A patient may experience two general types of health conditions: acute diseases and chronic diseases. Following the medical literature, acute conditions are defined as diseases that develop suddenly, are severe and sudden in onset (the initial phase of a disease or condition in which symptoms first become apparent), last a short time (often only a few days or weeks), and can be cured [34]. In contrast, a chronic disease is described as a human health condition or disease that is persistent or otherwise long-lasting in its effects or a disease that develops over time [35]. Thus, chronic diseases refer to long-term health conditions that last more than 1 year [36], whereas acute diseases refer to health conditions that are sudden, short-term, and require medical attention. Examples of acute diseases include the common cold, flu, and infections, whereas examples of chronic diseases include Alzheimer disease, arthritis, diabetes, and depression [36]. Given the contrasting nature of chronic and acute conditions, it is logical to argue that patients with different diseases will vary in their perceptions of AI in health care delivery. Previous research indicates that individuals tend to trust an algorithm or an AI system in low-risk conditions [37]. Based on that finding, we can expect those with acute short-term conditions but in severe pain to opt for an AI clinical encounter. For example, Wu and colleagues [38] explored older adults’ perceptions of mild cognitive impairment toward assistive AI and found a generally positive belief that these AI applications can be useful for the aging population.

#### Perceived Concerns and Risks

##### Perceived Communication Barriers

Conventionally, the health care delivery process usually occurs in a hospital or a physician’s clinic and involves direct physician-patient interaction, which can be described as paternalistic in nature. In other words, with medical expertise, the physician leads the patient toward shared medical decision-making, resulting in outcomes such as prescription and treatment plans centered on both evidence-based medicine and moral competency in terms of showing empathy and compassion. Empathy and compassion are increasingly being viewed as the foundations of active patient engagement and patient-centered care [39,40]. Research highlights that patients mainly seek and trust health care providers who are competent and compassionate with good interpersonal skills [41-44]. Researchers also reveal that empathetic and compassionate physician-patient interaction can improve patient satisfaction and greater clinical adherence [45]. Conversely, AI applications in service delivery (such as health care) may cause noteworthy communication barriers between customers and AI applications [46]. Reliance on AI clinical applications may reduce physicians’ and patients’ interactions and conversations [47]. Consumers may refuse to use AI applications because they need human social interaction during service encounters [10]. AI applications are powered with higher-level technical and evidence-based medicine but may not be expected to exhibit human-like empathy, which, in turn, may discourage patients from choosing the AI applications for the health care delivery process. Although the idea of building empathetic machines is well pursued in AI, patients’ perceptions toward the clinical encounters involving AI as substituting or augmenting technology versus traditional in-person visits warrant further investigation.

##### Perceived Transparency of Regulatory Standards

Physicians obtain their licensure after many rigorous training years in medicine and their specialty. This licensure is considered a regulating mechanism put forward by the government to ensure physicians’ quality and, ultimately, the quality of health care services. The licensure further allows a patient to choose a reliable doctor responsible for intentional errors or unintentional wrong-doings. However, in the AI context, regulatory authorities are yet to formalize standards to evaluate and maintain AI’s safety and impact in many countries [48]. Thus, people may become concerned if an appropriate regulatory and accreditation system regarding AI clinical applications is not yet in place. In the ever-changing AI and machine-learning landscape, more effective, efficient, and powerful algorithms are being developed on an everyday basis to power these AI health care applications. Often, the technical aspects of modern AI algorithms such as artificial neural networks (ANNs) remain a black box to society at large [49]. This is because after an ANN is trained with a data set, the quest to understand the algorithm’s decision-making process becomes essentially impossible. This perception of the “unknown” could potentially affect a patient’s preference for the clinical encounter. Furthermore, the lack of transparency in the regulatory standards that can be understood, critiqued, and reviewed [50] for AI applications by a larger community may discourage patients from choosing an AI encounter. The new IEEE (Institute of Electrical and Electronics Engineers) standard P7001 currently under development, “Transparency in autonomous systems,” has a set of measurable and testable levels of transparency that could be used to evaluate AI or autonomous systems for their level of compliance [51].

##### Perceived Liability Issues

A physician is usually held responsible for the consequences of their actions in a health care setting. With AI increasingly finding its way into health care research and practice, it becomes imperative to examine an AI system’s liability issues. Previous studies in public health demonstrate legal concerns about who will account for AI-based decisions when errors occur using AI applications [52]. Usually, the stakeholders in a medical encounter involving AI can be the developers, data feeders, health care organizations that adopted AI, or the health care provider that used the AI [53]. Noting that an AI application in itself cannot be held liable for any misdiagnosis or medical recommendations that turn out to be disastrous for a patient, the lack of standard consensus or regulations on who can be held liable may discourage patients from choosing an AI application. As AI clinical applications make autonomous decisions, the accountability question becomes very hard to answer. For instance, it will create a risky situation for both clinicians and patients when it is still unclear who becomes responsible if AI clinical applications offer wrong health care recommendations [54]. There is also no precise regulation regarding who is held liable when a physician follows the medical recommendations provided by AI and when a physician decides to override the recommendations [55].

##### Perceived Trust in AI Mechanisms, Collaborative Intelligence, and Physicians

Maintaining substantial trust between the public, health professionals, and health systems can create effective health care. Trust can be defined as trust in clinicians and the clinical tools they use (such as AI clinical applications) [48]. In the information systems (IS) literature, Vance and colleagues [56] call for additional research on trust in information technology artifacts such as AI systems. Gaining the general public’s trust in the use of AI in health care is considered an important challenge to the successful implementation of AI in medical practices [57]. Sun and Medaglia [58] reported that, in general, individuals are likely to exhibit a lack of trust in the features of AI systems. For instance, people may not trust AI’s predictive power and diagnostic ability for treatment purposes. Another study indicated that the autonomy of AI systems affects the users’ perception of trustworthiness [59]. Moreover, in a survey conducted by Longoni and Bonezzi [60] to understand customer perceptions about AI in medicine, only 26% of the sample signed up for an AI diagnosis compared to 40% who signed up for a health care provider diagnosis. In the same study, the authors found that the majority preferred a human provider over AI, even when it meant a higher risk of misdiagnosis. They also indicated that patients are more willing to choose AI if the ultimate treatment decision rests with the physician and not only the AI. These results highlight that individuals have a higher level of distrust toward AI in medicine than toward a human provider. Trust in AI clinical applications is a significant factor affecting adoption decisions [61]. Longoni et al [60] suggested that a physician’s confirmation of the AI results (an example of AI as augmenting technology) could encourage patients to be more receptive to AI in their care.

##### Perceived Performance Risks (Possible Errors)

AI-related studies consider the safety and quality of autonomous operations as essential factors affecting the use of AI applications [62]. According to Mitchell [63], AI applications are still vulnerable in many areas such as hacker attacks. Hackers can change text files or images, which may not have a human cognitive effect but could cause potentially catastrophic errors. Since the AI program may not understand the input and outputs, they are susceptible to unexpected errors and untraceable attacks. Performance risks may be serious in the context that directly deals with people’s lives (such as health care). Medical errors generated by AI could endanger patient safety and result in death or injuries, which are mostly not reversible. Thus, users may be concerned that the mechanisms used by AI clinical applications could lead to incorrect diagnoses or wrong treatments. Reddy et al [54] indicated that incomplete and nonrepresentative data sets in AI models can produce inaccurate predictions and medical errors. Thus, it could be expected that individuals may consider that possible functional errors resulting from using AI applications could lead to more risks.

##### Perceived Social Biases

Studies in other contexts have shown that AI models overestimate crime risk among members of a specific racial group [64]. In the health care context, biased AI models may overestimate or underestimate health risks in specific patient populations. For instance, AI applications may engage in stereotyping and exhibit gender or racial bias. Bias in AI models may also occur when data sets are not representative of the target population or when AI systems use incomplete and inaccurate data for decision-making [47]. Societal discrimination (such as poor access to health care) and small samples (such as minority groups) can lead to unrepresentative data and AI bias [48]. Edwards [65] argued that AI systems’ current architecture needs a more sophisticated structure to understand human moral values. If the AI algorithm is not transparent, it may exhibit some discrimination levels, even though humans are not involved in decision-making [66]. The main purpose of AI is to create an algorithm that functions autonomously to find the best possible solutions to questions [67]. However, researchers argue that predictive programs can be inevitably biased due to an overrepresentation of the social minorities in the pattern recognition process [68]. Some studies support this argument by showing that AI algorithms may be coded in a biased manner, which can produce racist decisions [69]. Therefore, if people are concerned that AI applications could lead to morally flawed health care practices by overestimating or underestimating health risks in a certain patient population, they will be more likely to perceive greater risks associated with AI.

##### Perceived Privacy Concerns

Health-related data are often viewed as constituting the most sensitive information about a person [47]. In health care services, respecting a person’s privacy is an essential ethical principle because patient privacy is associated with well-being and personal identity [70]. Thus, patients’ confidentiality should be respected by health care providers by protecting their health records, preventing secondary use of data, and developing a robust system to obtain informed consent from them for health care purposes [71]. If patients’ privacy needs are not met, patients will be affected by psychological and reputational harm [72]. Data breaches would increase risk beliefs associated with AI models designed to share personal health information. There is a concern that anonymized data can be reidentified through AI processes, and this anxiety may exacerbate privacy invasion and data breach risks [48]. AI applications in public health require large data sets. Thus, collecting, storing, and sharing medical data raise ethical questions about safety, governance, and privacy [73]. Privacy is one of the most critical concerns associated with using AI applications because users’ data (eg, habits, preferences, and health records) are likely to be stored and shared across the AI network [66]. The method of data collection for AI may increase risks as AI systems need huge data sets, and patients are concerned that their personal information will be collected without their knowledge [47].

##### Perceived Benefits

AI can be used in health care for risk prediction and recommendation generation. Big data and AI significantly improve patient health-based diagnosis and predictive capability [74]. Recent studies highlight new AI application opportunities within medical diagnosis and pathology, where medical tasks can be performed in an automated manner with higher speed and accuracy [75]. AI can improve health care delivery such as diagnostics, prognosis, and patient management [48]. For instance, AI has been shown to be capable of diagnosing skin cancer more efficiently than dermatologists [76]. Sohn and Kwon [77] demonstrated that hedonic aspects such as enjoyment and curiosity about AI technology are stronger in predicting the behavioral intention to use AI products than utilitarian aspects (eg, usefulness). This point does not hold in health care since AI applications are mainly used in health care for utilitarian aspects such as patient-specific diagnosis, treatment decision-making, and population risk prediction analysis [78]. Thus, with regard to benefit perceptions, in this study, we only focus on utilitarian aspects, not other motivational factors. Sun and Medaglia [58] proposed the lack of sufficient knowledge of the AI technologies’ values and advantages as potential barriers to adopting AI applications. Individuals will endorse and use AI clinical applications if they believe that AI will bring essential benefits to their health care delivery. Thus, we can expect that the higher the perceived benefits from AI clinical applications, the higher the individuals’ intention to use them in the future.

### Research Objectives

Most AI-related studies use various acceptance models (eg, technology acceptance model [TAM] and unified theory of acceptance and use of technology) to examine AI acceptance by empirically testing the effects of the ease of use, usefulness, and social norms on the intention to use AI applications [10,77]. For example, Xu and Wang [79] used the TAM to examine the adoption of AI robot lawyer technology for the legal industry. Another example is use of a TAM-based tool to measure AI-based assessment acceptance among students [80]. However, to the best of our knowledge, no experimental research has explored the differences in patients’ perceptions (with different types of illnesses) in relation to utilizing AI clinical applications without physician interactions, AI clinical applications with physician interactions, and traditional in-person visits. We hypothesized that the interactions between the type of health care service encounters and illness type may significantly change patients’ perceived risks, benefits, and overall attitudes toward health care delivery. The main objectives of this study were to: (1) examine the difference between the perceptions of patients with chronic diseases about utilizing AI clinical applications with and without physician interactions, and visiting physicians in person for diagnosis and treatment recommendation purposes; (2) investigate the difference between the perceptions of patients with acute diseases about utilizing AI clinical applications with and without physician interactions, and visiting physicians in person for diagnosis and treatment recommendation purposes; and (3) explore which service encounter is preferable for people with chronic diseases and that desired by people with acute diseases.

In this study, we focused on AI clinical applications for health care purposes. AI embedded in mobile health devices or health apps could help patients monitor their health status, check their health care information, and manage their chronic illnesses. These AI clinical applications use algorithms to learn from the past by analyzing the medical histories of patients with the same health conditions; recognizing patterns in clinical data; predicting possible health issues; and suggesting some treatment choices, diagnostic options, prescription advice, and care planning. These applications could reduce frequent patient-physician encounters and avoid unnecessary hospitalizations.

### Study Significance

This research offers significant and timely insight into human-computer interaction by examining AI applications in health care. This study’s findings will provide researchers and managers with critical insights into the determinants of individuals’ intention to use AI applications in health care delivery. The results imply that incompatibility with instrumental, technical, ethical, or regulatory values can be a reason for rejecting AI applications in health care. Multidimensional concerns associated with AI clinical applications may also be viewed as a cause of technostress, which occurs when an individual is unable to adapt to using technology [81]. In the future, it will be the patient’s or customer’s right to choose AI-driven recommendations over human care or vice versa. Nevertheless, we propose that AI application developers and programmers devise practical strategies to anticipate possible concerns and minimize risk beliefs to encourage individuals to use AI technology for health care purposes. Our results highlight that patients may have various reactions to AI (as substituting or augmenting) technology. Thus, different strategies and policies may need to successfully implement AI as a substituting or as an augmenting technology to address potential concerns and risks.

## Methods

### Study Design

**Figure 1:**
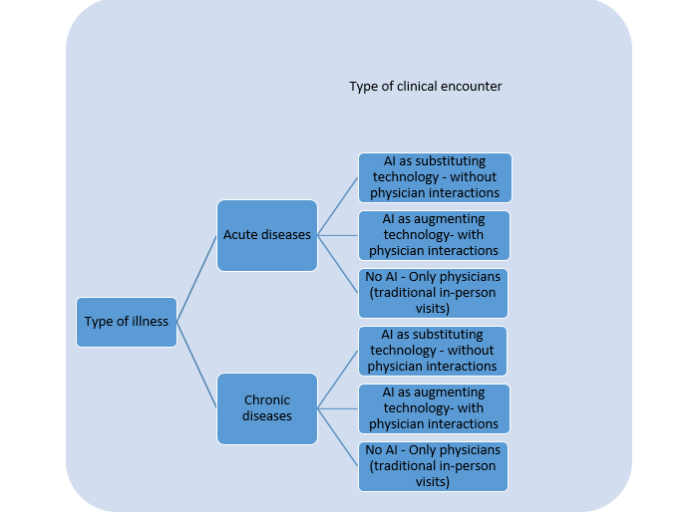
Six scenarios assigned to the study participants. AI: artificial intelligence.

To understand patients’ perceptions of AI applications in health care, we designed a 2×3 experiment that crossed a type of health condition (ie, acute or chronic) with three different types of clinical encounters (ie, AI clinical applications as substituting technology, AI clinical applications as augmenting technology, and no AI [traditional in-person visit]). In each scenario, we included two essential pieces of information: (1) the type of health condition and (2) the type of clinical encounter. We propose that the interactions between these two elements could lead to a comprehensive evaluation of how patients with different health conditions would perceive AI clinical applications in health care. It should be mentioned that participants of this study were actually suffering from either a chronic or acute disease. First, individuals entered their signs and symptoms to highlight what diseases they are suffering from. A filtering question was included at the beginning of the survey to categorize patients into the chronic or acute group. Each group was then randomly assigned to a hypothetical clinical encounter. For instance, an individual with an actual chronic illness was given a hypothetical situation in which they could use AI clinical applications under the physician’s control. Figure 1 illustrates the six scenarios resulting from two types of diseases and three types of clinical encounters.

In this study, we considered health conditions as either acute or chronic conditions. Acute diseases come on rapidly and are accompanied by distinct symptoms requiring urgent or short-term care, followed by improvement after treatment. For example, a broken bone that might result from a fall must be treated by a doctor and will heal in time. In some cases, an acute illness such as the common cold will simply go away on its own. Most people with acute illnesses will recover quickly. By contrast, chronic conditions develop slowly and may worsen over an extended period, from months to years. Chronic conditions are slower to develop, may progress over time, and may have many warning signs or no signs at all. Some examples of common chronic conditions are arthritis, diabetes, chronic heart disease, depression, high blood pressure, high cholesterol, and chronic kidney disease. Unlike acute conditions, chronic health conditions cannot be cured, only controlled and managed.

Regarding the second factor (clinical encounters), we focused on three categories: AI clinical applications as substituting technology, AI clinical applications as augmenting technology, and no AI (traditional in-person visit). In the AI as substituting technology scenarios (Scenarios 1-1 and 1-2), we defined a setting where patients directly use AI clinical applications for health care purposes. In these scenarios, we described a situation in which individuals can use an AI clinical application when they are suffering from a disease. The steps of using AI applications were clearly explained to respondents. For instance, when feeling sick, they can directly enter their signs, symptoms, and critical health complaints into the AI clinical application. Their health information will be recorded in a large database. The AI system then analyzes their health data, compares them to the learned patterns (eg, the list of diseases and medicines), and draws some clinical conclusions. Finally, based on the pattern found, the AI creates a report including some diagnostic options, some treatment choices, prescription advice (eg, dose, frequency and names of medications they need to take), care planning (eg, resting at home, taking suggested medicines for a specific period, or visiting a professional immediately). In summary, we highlighted that AI clinical applications could analyze clinical data and make medical decisions for patients without direct physician interactions. Therefore, we can consider this scenario using AI clinical applications without physician interactions to treat acute diseases or control chronic diseases.

In the AI as augmenting technology (with physician interaction) scenarios (Scenarios 2-1 and 2-2), we defined a setting in which patients have the option of using an AI clinical application that is monitored and controlled by their physician. In these scenarios, the physician will check the results and recommendations generated by the AI clinical application; discard some of the recommendations based on their experience and expertise; and make the final decision about the treatment choices, prescription options, and care planning. In summary, we emphasized that AI clinical applications can analyze clinical data and help physicians make medical decisions for patients. Thus, in this case, AI clinical applications are used with physicians’ direct supervision, and we can consider this scenario as AI-physician interactions (interactions and collaborations between physicians and AI).

In the no AI (only physician) scenarios (Scenarios 3-1 and 3-2), a patient has the option of visiting their physician. First, the physician asks for the patient’s signs, symptoms, and critical health complaints through a conventional in-person visit. The physician analyzes collected health information. Then, based on the physician’s knowledge, expertise, and experience, they draw some clinical conclusions. In summary, we highlighted that through a face-to-face patient-physician encounter, the physician can analyze clinical data and make final medical decisions. Thus, we can consider this scenario as an in-person visit with physicians. Table 1 displays the 2×3 experimental design of the six scenarios used in this study and the treatment group/scenario names.

**Table 1:** Research design overview.

Type of illness	AI^a^ as substituting technology without physician interaction	AI as augmenting technology with physician interaction	Traditional in-person visit
Acute: temporary, short-term diseases	Scenario 1-1: Acute-AI-only	Scenario 2-1: Acute-AI-physician	Scenario 3-1: Acute-physician-only
Chronic: long-lasting diseases	Scenario 1-2: Chronic-AI-only	Scenario 2-2: Chronic-AI-physician	Scenario 3-2: Chronic-physician-only

^a^AI: artificial intelligence.

Each experiment included three sections. First, the scenario was described, which detailed each experiment’s purpose (eg, in Scenario 1-2, the objective was defined as using AI clinical applications to control and monitor a chronic disease). Second, a set of questions about the nine outcome variables was provided to evaluate the respondents’ perceptions based on the given scenario. For example, subjects were asked to reflect on possible trust issues with AI applications (in Scenarios 1-1 and 1-2), with collaborative intelligence (in Scenarios 2-1 and 2-2), and with physician interactions (in Scenarios 3-1 and 3-2). Finally, we asked our subjects to provide some demographic information.

### Question Development

The main aim of this study was to evaluate individuals’ perceptions of the health care service options described by six scenarios. We used the following variables to measure patients’ perceptions: perceived performance risks, perceived communication barriers, perceived social biases, perceived privacy concerns, perceived trust, perceived transparency of regulatory standards, perceived liability issues, perceived benefits, and intention to use. We primarily included these variables to highlight the main barriers and facilitators of using AI clinical applications indicated by previous research [28]. Some variables such as perceived communication barriers and liability issues may have shared effects on the physician-patient interaction. However, in this study, we only focused on the impact perceived by patients. This study drew on the existing literature to measure the nine outcome variables used in the experiments, and minor changes were made to the questions to fit the given context (scenario). This study adapted items to measure outcome variables from existing scales developed by studies mainly conducted in the AI and medical fields. The descriptions of scenarios and final measure items used in this study are listed in Multimedia Appendix 1. Table 2 shows the definitions of all outcome variables used in this study.

**Table 2:** Operationalization of outcome variables.

Outcome variables	Variable definition	Reference
Perceived performance risks	The degree to which an individual believes that the clinical encounter (which is explained in the scenario) will exhibit pervasive uncertainties	Marakanon and Panjakajornsak [82]
Perceived communication barriers	The degree to which an individual feels that the clinical encounter (which is explained in the scenario) may reduce human aspects of relations in the treatment process	Lu et al [46]
Perceived social biases	The degree to which a person believes that a clinical encounter (which is explained in the scenario) may lead to societal discrimination to a certain patient group (eg, minority groups)	Reddy et al [48]
Perceived privacy concerns	The extent to which individuals are concerned about how the clinical encounter (which is explained in the scenario) will collect, access, use, and protect their personal information	Zhang et al [83]
Perceived trust	The degree to which an individual believes that the clinical encounter (which is explained in the scenario) is trustworthy	Luxton [55]
Perceived transparency of regulatory standards	The extent to which an individual believes that regulatory standards and guidelines to assess the safety of the clinical encounter (which is explained in the scenario) are yet to be formalized	Cath [70]
Perceived liability issues	The extent to which an individual is concerned about the liability and responsibility of using the clinical encounter (which is explained in the scenario)	Laï et al [20]
Perceived benefits	The extent to which an individual believes that the clinical encounter (which is explained in the scenario) can improve diagnostics and care planning for patients	Lo et al [84]
Intention to use	The extent to which an individual is willing to use the proposed clinical encounter (which is explained in the scenario) for diagnostics and treatments	Turja et al [27]

Since this study’s subjects were individuals, we took two steps to ensure that the definitions, given scenarios, and questions were illustrated to be understandable for the general public. First, once the initial scenarios and surveys were developed, we consulted three professionals in the AI domain and two physicians (who were familiar with AI clinical applications) to improve our study’s content validity and finalize the definitions, scenarios, and questions used in each survey. Consistent with the experts’ suggestions, we modified the terms used to describe AI clinical applications, AI-physician interaction, as well as in-person examination, and improved the scenarios and questions to ensure that they were sufficiently transparent and easy to understand for the public. Second, we performed a face validity evaluation with 14 students (2 doctoral students in computer science, 1 doctoral student in IS, 4 master’s students in computer science, 5 master’s students in IS, and 2 medical students) to ensure that the readability of the scenarios and wording of the questions were acceptable and consistent with the objectives of our study. Thus, we reworded some ambiguous terms and removed technical language and jargon to describe the scenarios and develop the surveys in an understandable manner. It should be mentioned that graduate students may have a higher reading level than an average person. However, they were asked to detect and flag technical expressions and ambiguous terms that might not be clear to an average person. Therefore, the graduate students used more scrutiny to focus on every detail to ensure the questions were sufficiently transparent for our potential sample.

### Data Collection

This study was reviewed and approved by the Institutional Review Board of Florida International University, and the data collection was performed confidentially. Written informed consent was obtained from all participants. All methods used in this study were carried out in accordance with relevant guidelines and regulations.

We used a power analysis to identify the appropriate sample size per scenario. The results of the power analysis showed that for a range of medium (0.5) to high (0.8) effect size [85], with α=.05 and power of more than 0.8, the total minimum sample required is about 50 respondents per scenario. In this study, there were nine main outcome variables with 49 measures. Therefore, to reduce possible sampling errors, we initially collected a sample of 121 respondents per scenario to ensure an adequate sample size after data cleaning and matching respondents in different scenarios. Data were collected in May 2020 from Amazon’s Mechanical Turk (MTurk) to obtain a representative group of subjects in the United States. MTurk is a survey tool used in previous research that is considered as an acceptable means to collect individual-level data from the general population of interest [86]. The surveys of six scenarios were posted to MTurk, and the respondents’ location was limited to the United States. We enabled a microcode in the survey design to prevent respondents from taking each survey more than once. Following previous studies that used MTurk for data collection, a monetary reward (US $0.70) was given as the incentive for participation. The range of average completion time for the six experimental groups was between5 minutes, 17 seconds and 8 minutes, 49 seconds, which indicated acceptable responses in terms of the time spent on each survey by the participants.

### Data Analysis

IBM SPSS Statistics V21.0 was used to analyze the data. Propensity score matching was used with a tolerance of 0.05 to match participants and avoid any demographic bias between scenarios. To find each outcome variable’s total score, we calculated unweighted sum scores of items for each variable. Analysis of variance (ANOVA) was then performed to examine the differences between the six proposed scenarios for each of the outcome variables: perceived performance risk, perceived social biases, perceived privacy concerns, perceived trust, perceived communication barriers, perceived concerns about the transparency of regulatory standards, perceived liability issues, perceived benefits, and intention to use. Prior to ANOVA, the Levene test was run to ensure the homogeneity of variance, as this is one of the fundamental assumptions of ANOVA. There was sufficient evidence to hold the assumption of homogeneity of variance for all outcome variables. The Scheffe posthoc test was used to identify which scenarios significantly differed from each other per outcome variable.

## Results

After cleaning the data for biases and incomplete responses, there were a total of 634 completed surveys. After matching across scenarios, there were 105 participants in Acute-AI-only, 104 participants in Chronic-AI-only, 113 participants in Acute-AI-physician, 103 participants in Chronic-AI-physician, 105 participants in Acute-physician-only, and 104 participants in Chronic-physician-only. The detailed demographic information of the six scenarios is reported in Multimedia Appendix 2. In summary, 44% of participants were women; approximately 30% of participants were between 20 and 29 years old, 33% were between 30 and 39 years old, 18% were between 40 and 49 years old, and 17% were above 50 years of age. The majority of the participants were White (65%), followed by 17% Asian, 11% African American, and 5% Hispanic. Regarding the level of education, 6% were high school graduates, 11% completed some college, 8% held a 2-year degree, 48% had a bachelor’s degree, and 23% had a master’s degree. Regarding employment status, most of the participants were full-time employees (72%), followed by 14% part-time employees, 8% unemployed, 2% retired, and 4% students. Approximately 15% of participants in our study reported an annual household income of less than US $25,000, 26% reported an income between US $25,000 and US $49,999, 22% reported an income between US $50,000 and US $74,999, 18% reported an income between US $75,000 and US $99,999, and approximately 19% reported an income of more than US $100,000.

Across the six scenarios, there were no significance differences in terms of gender *(χ^2^*_5_=1.76, *P*=.88), age (*χ^2^*_25_=30.31, *P*=.21), race (*χ^2^*_25_=22.37, *P*=.62), level of education *(χ^2^*_30_=37.89, *P*=.15), employment (*χ^2^*_20_=16.20, *P*=.70), and annual household income (*χ^2^*_25_= 19.85*, P*=.76). Respondents were also asked to report their personal innovativeness on a Likert scale to ensure this factor would not introduce any bias into different scenarios. The ANOVA results across the six scenarios revealed no significant differences among respondents regarding the level of personal innovativeness (*P*=.19).

The items were adapted from previous studies with slight changes to fit them into this research context. All items were measured on a 5-point Likert-type scale, with 1 indicating “strongly disagree” and 5 indicating “strongly agree.” Table 3 shows the number of items and Cronbach α values per outcome variable, which were all above .70 as the recommended threshold value [87], implying adequate reliability per outcome variable.

**Table 3:** Reliability of variables.

Outcome variables	Number of items	Cronbach α
Perceived performance risks	5	.92
Perceived social biases	4	.85
Perceived privacy concerns	6	.93
Perceived trust	5	.92
Perceived communication barriers	5	.92
Perceived transparency of regulatory standards	5	.92
Perceived liability issues	6	.93
Perceived benefits	7	.92
Intention to use	5	.92

The summary statistics (mean score, SD) per outcome variable are presented in Table 4. Some of the trends are evident from these results. For example, we can observe lower privacy concerns and liability issues for traditional in-person examinations than AI-based interactions.

**Table 4:** Summary statistics of outcome variables as a function of a 2 (type of illness) by 3 (type of encounter) design.

Outcome variable		AI^a^ as substituting technology (without physician interaction), mean (SD)	AI as augmenting technology (with physician interaction), mean (SD)	Traditional in-person visit, mean (SD)	ANOVA^b^			
					*F* statistic (*df*=5, 628)		*P* value	
**Perceived performance risks**					1.36		.24	
	Acute, short-term illness	16.3 (5.1)	16.6 (5.5)	15.4 (5.0)				
	Chronic, long-lasting illness	16.2 (5.1)	17.1 (4.8)	15.9 (5.3)				
	Marginal means^c^	16.3 (5.1)	16.8 (5.1)	15.6 (5.1)				
**Perceived biases**						0.86		.51
	Acute, short-term illness	12.8 (3.7)	13.0 (4.4)	12.2 (3.8)				
	Chronic, long-lasting illness	13.0 (3.9)	13.2 (3.8)	12.4 (4.3)				
	Marginal means	12.9 (3.8)	13.1 (4.1)	12.3 (4.0)				
**Perceived privacy concerns**						3.35		.005
	Acute, short-term illness	19.0 (6.7)	20.8 (6.1)	17.7 (6.1)				
	Chronic, long-lasting illness	20.5 (5.8)	19.8 (6.1)	19.5 (7.0)				
	Marginal means	19.8 (6.3)	20.3 (6.1)	18.6 (6.6)				
**Perceived trust**						6.27		<.001
	Acute, short-term illness	16.5 (5.0)	17.3 (4.8)	18.6 (4.4)				
	Chronic, long-lasting illness	15.4 (4.9)	16.8 (4.7)	18.2 (4.8)				
	Marginal means	16.0 (5.0)	17.1 (4.8)	18.4 (4.6)				
**Perceived communication barriers**						9.24		<.001
	Acute, short-term illness	17.4 (5.7)	18.1 (5.2)	14.7 (4.8)				
	Chronic, long-lasting illness	17.1 (4.9)	17.5 (4.8)	14.6 (5.7)				
	Marginal means	17.3 (5.3)	17.8 (5.0)	14.6 (5.3)				
**Perceived transparency of regulatory standards**						9.42		<.001
	Acute, short-term illness	17.9 (5.0)	18.1 (5.1)	14.9 (4.9)				
	Chronic, long-lasting illness	17.6 (4.7)	17.6 (5.0)	15.0 (5.5)				
	Marginal means	17.8 (4.9)	17.8 (5.0)	14.9 (5.2)				
**Perceived liability issues**						6.27		<.001
	Acute, short-term illness	21.3 (6.4)	22.1 (5.7)	18.5 (6.0)				
	Chronic, long-lasting illness	20.8 (5.8)	20.6 (6.2)	18.3 (6.7)				
	Marginal means	21.0 (6.1)	21.4 (6.0)	18.4 (6.4)				
**Perceived benefits**						3.28		.006
	Acute, short-term illness	24.5 (6.2)	25.9 (5.5)	26.1 (5.9)				
	Chronic, long-lasting illness	23.6 (6.5)	24.2 (6.6)	26.0 (6.2)				
	Marginal means	24.1 (6.4)	25.1 (6.1)	26.1 (6.0)				
**Intention to use**						9.71		<.001
	Acute, short-term illness	16.6 (4.8)	17.4 (4.9)	19.3 (4.6)				
	Chronic, long-lasting illness	15.8 (5.3)	16.5 (5.2)	19.3 (4.6)				
	Marginal means	16.2 (5.1)	17.0 (5.0)	19.3 (4.6)				

^a^AI: artificial intelligence.

^b^ANOVA: analysis of variance.

^c^Difference in the means of acute short-term illness and chronic long-lasting illness.

Significant differences (*P*<.05) between different groups were found for the following variables: perceived privacy concern, perceived trust, perceived communication barriers, perceived concerns about transparency in regulatory standards, perceived liability issues, perceived benefits, and intention to use. No significant difference was found between scenarios regarding perceived performance risk and perceived social biases.

Table 5 shows a summary of significant differences between scenarios from the Scheffe posthoc test. Patients suffering from an acute temporary short-term disease were significantly more concerned about the privacy of their health information when AI clinical applications with physician interaction was used compared to having a traditional in-person interaction with their physicians (*P*=.03). No significant differences were found in terms of perceived privacy concerns among patients with chronic conditions across scenarios.

Concerning trust, our results showed that patients with chronic illnesses found AI clinical applications to be less trustworthy compared to traditional diagnostic and treatment processes when they interact directly with the physicians (*P*=.004). The trust in physicians was also significantly higher for patients with acute conditions (*P*<.001).

Regarding perceived communication barriers, patients were significantly more concerned that AI clinical applications may reduce or eliminate the human aspect of relations between patients and professional care providers in comparison with face-to-face physician interactions for both acute (*P*=.01) and chronic (*P*<.001) health conditions. Similarly, when AI clinical applications are used in addition to physician interaction, there were still significantly greater concerns about lack of human relations than in face-to-face physician visits for both acute (*P*=.03) and chronic (*P*=.005) illnesses.

Further, the results showed that patients were significantly more concerned about the transparency of regulatory standards to assess AI algorithms and tools in comparison with the transparency of guidelines to monitor the performance of physicians’ practices for both acute (*P*=.002) and chronic (*P*=.02) conditions. Similarly, when AI clinical applications are used in addition to physician interactions, patients were significantly more concerned about the transparency of guidelines for AI-physician interactions than traditional in-person physician visits for acute (*P*=.001) and chronic (*P*=.02) illnesses.

Regarding patients’ concerns about liability issues, patients with acute illnesses were significantly more concerned when AI clinical applications are used under physicians’ control. This may be because of the lack of clarity about who is responsible if appropriate AI-recommended treatment options are mistakenly dismissed or offer wrong recommendations compared to physician liability in traditional visits (*P*=.003). Interestingly, patients suffering from a chronic condition were significantly more concerned about liability issues using only AI clinical applications (*P*=.04) or AI tools with physician control (*P*=.001).

Lastly, patients with acute illnesses indicated significantly higher intentions to use in-person visits than only AI clinical applications (*P*=.01). By contrast, patients with chronic illnesses were significantly more willing to use in-person visits compared to only AI tools (*P*<.001) as well as AI clinical applications under physician control (*P*=.006). The detailed results, including nonsignificant differences, are included in Multimedia Appendix 3.

**Table 5:** Comparison of outcome variables between the six scenarios.

Scenarios compared			Mean difference (SE)	*P* value		95% CI
**Perceived privacy concern**						
	Acute-AI-physician vs Acute-physician-only	3.07 (0.86)		.03	0.21 to 5.92	
**Perceived trust**						
	Chronic-AI-only vs Acute-physician-only	–3.22 (0.66)		<.001	–5.42 to –1.01	
**Perceived communication barriers**						
	Acute-AI-only vs Acute physician only	2.75 (0.72)		.01	0.36 to 5.14	
	Acute-AI-only vs Chronic-physician-only	2.86 (0.72)		.01	0.46 to 5.26	
	Chronic-AI-only vs Acute-physician-only	2.41 (0.72)		.05	0.01 to 4.81	
	Chronic-AI-only vs Chronic physician-only	2.52 (0.72)		.03	0.12 to 4.92	
	Acute-AI-physician vs Acute-physician-only	3.38 (0.70)		<.001	1.03 to 5.73	
	Acute-AI-physician vs Chronic-physician only	3.49 (0.71)		<.001	1.13 to 5.84	
	Chronic-AI-physician vs Acute-physician-only	2.87 (0.72)		.01	0.46 to 5.27	
	Chronic-AI-physician vs Chronic-physician-only	2.98 (0.72)		<.001	0.57 to 5.38	
**Perceived transparency of regulatory standards**						
	Acute-AI-only vs Acute-physician-only	3.04 (0.70)		<.001	0.71 to 5.36	
	Acute-AI-only vs Chronic-physician-only	2.91 (0.70)		<.001	0.57 to 5.24	
	Chronic-AI-only vs Acute-physician-only	2.77 (0.70)		.01	0.44 to 5.10	
	Chronic-AI-only vs Chronic-physician-only	2.64 (0.70)		.02	0.30 to 4.97	
	Acute-AI-physician vs Acute-physician-only	3.22 (0.68)		<.001	0.94 to 5.51	
	Acute-AI-physician vs Chronic-physician-only	3.09 (0.69)		<.001	0.80 to 5.38	
	Chronic-AI-physician vs Acute-physician-only	2.71 (0.70)		.01	0.37 to 5.04	
	Chronic-AI-physician vs Acute-physician-only	2.57 (0.70)		.02	0.23 to 4.91	
**Perceived liability issues**						
	Acute-AI-only vs Chronic-physician-only	2.92 (0.85)		.04	0.09 to 5.75	
	Acute-AI-physician vs Acute-physician-only	3.53 (0.83)		<.001	0.75 to 6.30	
	Acute-AI-physician vs Chronic-physician-only	3.73 (0.83)		<.001	0.94 to 6.51	
**Intention to use**						
	Acute-AI-only vs Acute-physician-only	–2.75 (0.68)		.01	–5.02 to –0.49	
	Acute-AI-only vs Chronic-physician-only	–2.73 (0.68)		.01	–5.00 to –0.46	
	Chronic-AI-only vs Acute-physician-only	–3.52 (0.68)		<.001	–5.79 to –1.25	
	Chronic-AI-only vs Chronic-physician-only	–3.49 (0.68)		<.001	–5.76 to –1.22	
	Chronic-AI-physician vs Acute-physician-only	–2.79 (0.68)		.01	–5.06 to –0.52	
	Chronic-AI-physician vs Chronic-physician-only	–2.76 (0.68)		.01	–5.04 to –0.48	

## Discussion

### Principal Findings

Given the promising opportunities created by AI technology (such as better diagnostic and decision support), the main question is when AI applications will become part of routine clinical practice [88]. AI embedded in smart devices democratizes health care by bringing AI clinical applications into patients’ homes [47]. Nevertheless, some concerns related to the use of AI need to be addressed. As previous studies introduced several concerns and challenges with AI [66], this study’s main focus was to analyze the perceptions of people with different health conditions about the use of AI clinical applications as an alternative for diagnostics and treatment purposes. This study required participants who are actually suffering from an acute or chronic disease to consider hypothetical situations (ie, using AI clinical applications). If the study had recruited participants who are current users of AI clinical applications for health care purposes, the results could have been different. Current users of AI applications in health care settings may have more accurate perceptions about AI, and the findings could be more practical. In the following subsections, we propose our theoretical contributions and practical implications related to each outcome variable.

### Perceived Communication Barriers

The results showed that people with both acute and chronic health conditions may believe that both AI applications and collective intelligence can lead to communication barriers. This point is in line with previous studies highlighting that the use of AI applications in service delivery (such as health care) may cause noteworthy communication barriers between customers and service providers [46]. Reliance on AI clinical applications may reduce physicians’ and patients’ interactions and conversations [47]. Consumers may refuse to use AI applications because they need human social interaction during service encounters [10]. AI technology fundamentally changes traditional physician-patient communications. Thus, individuals may worry as they may lose face-to-face cues and personal interactions with physicians. AI creates challenges to patient-clinician interactions, as clinicians need to learn how to interact with the AI system for health care delivery and patients are required to reduce their fear of technology [89]. As AI continues to proliferate, users still encounter some challenges concerning effective use, such as how the partnership between AI systems and humans could be synergic [2]. A previous study proposed that more sophisticated technologies should be integrated into current AI clinical applications to improve human-computer interactions and streamline the information flow between two parties [66]. Therefore, the nature of AI clinical applications (even coupled with physician controls) may reduce conversation between physicians and patients, resulting in the emergence of more risk beliefs.

Suppose individuals are concerned that AI applications may reduce human aspects of relations in medical contexts. In that case, they may lose face-to-face cues and personal interactions with physicians and find themselves in a more passive position for making health-related decisions. This finding is consistent with a study in the chatbot context (within the area of AI systems), which indicated that users have stronger feelings of copresence and closeness when the chatbot uses social cues [90]. In the context of robot care, a study showed that when robots are used in rehabilitation, they are viewed by patients as reducing human contact [91]. Developers need to add more interactive and entertaining social cues to AI clinical applications to address the possible communication barriers between users and AI. For instance, AI-driven recommendations and assistance can be appealing if the application holds promise of allowing users more time to interact with it to establish empathy.

### Perceived Privacy Concerns

In the case of suffering from an acute illness, people may perceive more serious privacy concerns if they can use AI clinical applications that are under physician control. Thus, they may prefer to use face-to-face interactions with physicians to reduce their privacy concerns. Deeper privacy concerns may have roots in two common perceptions. The first is the belief that anonymized data can be reidentified through AI models, and in turn, could increase the likelihood of privacy invasion and data breach [48]. The second is that AI systems need massive data sets; thus, patients are concerned that their health information may be collected or shared without permission for purposes other than treatment [47].

### Perceived Trust

The findings imply that individuals with chronic conditions may not trust AI clinical applications if no physician interactions are included in health care delivery. According to previous studies, the nature of AI models (such as deep learning) may increase a lack of transparency related to AI systems and threaten patient trust, resulting in higher risk beliefs [57]. When patients cannot understand the inside workings of AI applications (such as decision-making models), they may exhibit lower trust in their functions and how they generate treatment solutions and recommendations. Thus, people with chronic diseases may be more willing to trust direct patient-physician interactions to control and manage their symptoms.

### Perceived Accountability Issues

Accountability and liability are the other major concerns related to the use of AI. In this study, patients with acute conditions were more likely to be concerned about liability issues in the scenarios of both purely AI clinical applications and AI-physician interactions. Acute diseases are often accompanied by distinct symptoms that require urgent or short-term care. Thus, patients with severe and sudden signs and symptoms may seek quick care planning, accurate diagnosis, and reliable treatment options to cure their health problems promptly. In this situation, patients will become more nervous if they do not know who is held responsible for possible medication errors (such as wrong drug selection, wrong dose, or wrong quantity). This finding is consistent with previous studies in public health demonstrating the legal concerns surrounding who can be held accountable for AI-based decisions when errors occur using AI systems [52]. In general, society is yet to fully grasp many of the accountability and responsibility considerations associated with AI and big data [92]. Accountability involves several stakeholders such as AI developers, government agencies, health care institutions, health care professionals, and patient communities. Nevertheless, it is still not clear how the regulatory concerns around responsibility and accountability of using solutions made by AI systems can be dealt with formally [66]. Liability complexity becomes higher since it is not transparent to what extent AI systems can guide and control clinical practices [93]. Responsibility concerns are not only limited to the incidents in which AI may generate errors. Another aspect of liability risk is when the right and appropriate treatment options recommended by AI are mistakenly dismissed [55]. Thus, the higher the perceived liability issues, the greater the risk beliefs associated with AI. Regulatory agencies and health care organizations require clear policies to identify each stakeholder’s responsibility (eg, patients, physicians, hospitals, and AI developers) when AI clinical applications are widely offered.

### Perceived Transparency of Regulatory Standards

Regarding the regulatory risks associated with the transparency of standards, patients with either acute or chronic conditions were concerned with using purely AI-based services as well as AI applications under physicians’ direct supervision. This is in line with previous studies, which highlight that regulatory concerns are critical challenges to the use of AI in health care as the policies and guidelines for AI applications are not yet transparent [70]. The existing literature indicates that regulatory agencies require agreement on a set of standards that medical AI rollout must be rated against, such as determining the reliability of auditing the decisions made by autonomous AI clinical applications [66]. Due to the intelligence nature of AI systems, regulatory agencies should establish new requirements, official policy, and safety guidelines regarding AI rollout in health care [20]. For example, there is a legal need to evaluate the decision made by AI systems in case of litigation. AI applications operate based on autolearn models, which improve their performance over time [94]. This inner mechanism differentiates AI applications from other health care tools and gives rise to new regulatory concerns that may not be the case in different domains. Generally, algorithms that change continuously with features that are not limited to the original accepted clinical trials may need a new range of policies and guidelines [47]. Regulatory authorities are yet to formalize standards to evaluate and maintain AI’s safety and impact in many countries [48]. Thus, people may become concerned if an appropriate regulatory and accreditation system regarding AI clinical applications is not yet in place.

The lack of clear guidelines to monitor the performance of AI applications in the medical context can lead to higher risk beliefs associated with AI. Hence, if health care organizations cannot reduce regulatory concerns, many individuals may refuse to use AI clinical applications and request traditional interactions with physicians. Even if hospitals decide to use AI applications as supportive services under health care professionals’ supervision, the regulatory concerns should be mitigated prior to implementing AI systems. Regulatory agencies should establish normative standards and evaluation guidelines for implementing and using AI in health care in cooperation with health care institutions. The policies should clarify how AI clinical applications will be designed and developed in health care to comply with the accepted ethical principles (such as fairness and health equity). Regular audits and ongoing monitoring and reporting systems can be used to continuously evaluate the safety, quality, transparency, and ethical factors associated with services delivered through AI clinical applications.

### Perceived Performance Risks

There were no significant differences found across the scenarios regarding performance risks. This result may reflect the belief of people with either acute or chronic diseases that the possibility of making medical errors with AI clinical applications would be the same as that for traditional in-person visits with physicians, even when doctors monitor the AI applications. Thus, the findings provide no solid evidence that individuals may believe that AI models and their features exhibit functional errors or technological uncertainties that endanger patient safety and lead to death or injuries. Respondents reported that any clinical encounters (ie, traditional, collaborative intelligence, or AI applications) could lead to incorrect diagnoses or wrong treatments.

### Perceived Benefits

The results demonstrated significant differences in the perceived benefits according to the scenarios when using three alternative clinical encounters. Although the posthoc test did not show a significant difference among the six experimental groups, we observed that people with either acute or chronic conditions associated more benefits to direct interactions with their physicians. Among the AI options, only Acute-AI-physician (acute conditions and collaborative intelligence) showed similar responses to those given for traditional face-to-face interactions. Moreover, the scores of Acute-AI-physician on perceived risks such as privacy concerns, communication barriers, as well as regulatory and liability issues were not significantly different from those of other AI-based scenarios. Therefore, since the Acute-AI-physician scenario was associated with relatively higher benefits and nonsignificant differences in risk perceptions, we can argue that it might be a better option to start with. Accordingly, we recommend that implementing an AI-based service that physicians directly control and monitor would be an acceptable choice for patients with acute diseases.

Moreover, these results suggest that health care organizations, physicians, and AI application developers need to highlight potential AI benefits in their marketing campaigns to promote usability and the value of their AI applications, and ultimately increase the rate of usage. This argument is consistent with other studies suggesting that patients become more likely to use AI clinical applications if they believe they can improve diagnostics, prognosis, and patient management systems [95]. Specific marketing strategies in medical AI application companies and hospitals can be developed to enhance users’ awareness about both human and computer intelligence. These strategies should enlighten physicians on maintaining interactions with patients while using AI clinical applications that could suggest accurate care planning, reduce health care costs, and boost health care outcomes. Thus, highlighting the performance benefits of AI, such as accuracy of diagnosis, reliability of data analysis, the efficiency of care planning, and consistency of treatments, in communication with users along with marketing materials may increase individuals’ intention to at least try services provided by AI applications in health care.

### Intention to Use

The results indicated that people with chronic diseases are less willing to solely use AI clinical applications or AI applications controlled by physicians. Since chronic conditions encourage patients to visit their physicians frequently to consult them about their illness signs, symptoms, and progress, they are generally more likely to prefer human-human consultations over human-computer interactions. This point highlights that patients suffering from a long-lasting disease may not be ready to use pure or partial AI clinical applications to control their chronic conditions. Therefore, health care organizations need to exercise caution when implementing these applications for chronic diseases.

Furthermore, it should be mentioned that even though one of the primary outcome variables in this study was the intention to use, we do not propose that an unconditional acceptance of AI clinical applications is the ideal situation in health care. In contrast, we exhibit how important value-based consideration is when implementing AI applications in health care contexts. Suppose the rejection of medical AI is explained by huge and unaddressed technological, ethical, or regulatory concerns. In that case, there is not much sense in partially coping with these concerns by setting up the mandatory use of medical AI covering the entire patient spectrum. We propose that a successful rollout of AI clinical applications be managed with the knowledge and consideration of potential users’ benefits and risk perceptions. There is growing interest in research about AI-centric technologies; however, individuals have not yet integrated AI applications into many aspects of their lives [96]. We can argue that the public’s general technical knowledge about AI performance and how it works is still at an early stage. If AI clinical applications gained more ground in everyday care work, people would have a better perspective on the benefits and risks associated with them and actually start using them.

### The Role of Training

AI clinical applications should be designed in a way to respect patients’ autonomy and decision-making freedom. AI agents should not follow a coercive approach to force patients to make health-related decisions under pressure. Regulations should illuminate patients’ roles in relation to AI applications so that they are aware of their position to refuse AI-based treatments where possible [97]. An important aspect that needs to be built into AI systems in health care is the transparency of AI algorithms so that the AI system does not remain a black box to the users. Technical education, health knowledge, and explicit informed consent should be emphasized in the AI implementation model to prepare patients for AI use. Training should target the patient community to ensure that the patients obtain sufficient information to make informed health decisions. Thus, if users understand the basics of AI applications, and the potential benefits and limitations they can bring to health care, they will become more willing to accept AI use to obtain improved health care delivery. Under this circumstance, users will be active partners of AI applications rather than passive AI recommendation receivers.

### Limitations and Future Work

Although this study provides theoretical and practical implications, it has some limitations. First, we collected data from a sample of respondents from the United States. Care work culture and technology use are diverse among different countries. Therefore, we recommend that future studies consider subjects from other geographical locations such as other developed countries and developing countries that may not yet be implementing and using technologically advanced infrastructures in health care services (such as smart devices or AI clinical applications). Second, our study used an online survey to recruit participants digitally, and several measures were taken to provide clear definitions and scenarios. Since a self-rated sample of participants on MTurk was used, there is still a small chance that some respondents were not completely aware of AI technology and may have formed their own perceptions of the information technology artifact. Therefore, we suggest that further studies use a different method to ensure that subjects are knowledgeable about medical AI. For instance, future research can recruit informed patients who are directly referred by the providers using patient self-management tools such as wearable devices with embedded AI.

Third, due to the online data collection procedure through MTurk, we only considered respondents who could access the internet and were healthy enough to participate in an online survey. Although the MTurk pool has been recognized as an acceptable data collection means for academic research, caution should be exercised when generalizing this study’s results. Future researchers may extend this study by using other data collection methods to reach out to patients. Since the experiments did not occur within a health care setting (such as a hospital), the generalizability of our findings could have been limited. Thus, it would be interesting for future studies to repeat the same experiments through simulations with treating physicians and patients suffering from acute and chronic diseases visiting a health care center (eg, a hospital).

Fourth, the lack of educational background diversity (eg, 71% of participants had higher education) and age variation (eg, 63% were younger than 40 years) of the sample may be considered a limitation for the generalizability of our results. Thus, it is recommended that future studies consider drawing samples with more representative subjects in wider age groups with various levels of education. Fifth, we used the general concept of AI, and no specific type of AI clinical application was examined. Users’ perceptions may have different underlying objectives depending on the type of AI application considered. However, this study can also serve as a starting point for further empirical studies in the context of individual adoption of AI clinical applications. For instance, it would be interesting to investigate how alternative AI application brands influence risk beliefs, perceived benefits, and intention to use. Finally, we defined AI applications as tools that consumers can voluntarily choose to use for health care management. Another promising research avenue would be to examine public perspectives in other health care contexts such as when AI applications are implemented and used in hospitals and health care professionals recommend that patients start to use these applications. We also recommend a follow-up study examining users’ value perceptions in the context of mandatory AI applications used for diagnosing and completing patient treatments.

### Conclusions

Disruptive advances in technology inevitably change societies, communications, and working life. Technology and health care have become inseparable in recent times. One of the fundamental technological changes that could impose significant health care effects is the widespread implementation and use of AI clinical applications. AI technology is an integral element of many organizations’ business models, and it is a critical strategic component in the plans for many sectors of business such as health care institutions. Implementing advanced information systems (such as AI) in health care requires an in-depth understanding of the factors associated with technology acceptance among groups of stakeholders. One of the most important stakeholders of AI clinical applications is patients. Due to the distinct characteristics of the health care sector, the implementation of AI applications should be conducted with several necessary considerations. From the public perspective, using AI applications is a form of endorsing them. Our results highlight that there are still noticeable concerns about implementing AI clinical applications in diagnostics and treatment recommendations for patients with both acute and chronic illnesses, even if these tools are used as a recommendation system under physician experience and wisdom. Our study shows that individuals may still not be ready to accept and use AI clinical applications owing to some risk beliefs. Before implementing AI, more studies are needed to identify the challenges that raise concerns for the implementation and use of AI tools. We recommend addressing the concerns contributing to risk beliefs about using AI clinical applications as a priority for health care organizations. If privacy concerns, trust issues, communication barriers, concerns related to the transparency of regulatory standards, and liability risks are not analyzed, rationalized, and resolved accordingly, people may not use these applications. They may further view AI applications as a threat to their health care. AI application developers and health care providers need to highlight the potential benefits from AI technology and address different dimensions of concerns to justify using an AI clinical application to the public. Health care regulatory agencies need to clearly define the rights and the responsibilities of health care professionals, developers, programmers, and end users to demonstrate acceptable approaches in using AI applications in health care.

## Appendix

Multimedia Appendix 1 Experiments and scenarios.

Multimedia Appendix 2 Demographic information of participants in the six scenarios.

Multimedia Appendix 3 Detailed Scheffe posthoc test results.

## Abbreviations

AI: artificial intelligence
ANN: artificial neural network
ANOVA: analysis of variance
FST: frontline service technology
IEEE: Institute of Electrical and Electronics Engineers
IS: information systems
MTurk: Mechanical Turk (Amazon)
TAM: technology acceptance model

## References

[1] Kaplan A, Haenlein M (2019). Siri, Siri, in my hand: Who’s the fairest in the land? On the interpretations, illustrations, and implications of artificial intelligence. Bus Horiz.

[2] Jarrahi MH (2018). Artificial intelligence and the future of work: human-AI symbiosis in organizational decision making. Bus Horiz.

[3] Bitner MJ, Brown SW, Meuter ML (2000). Technology infusion in service encounters. J Acad Mark Sci.

[4] Marinova D, de Ruyter K, Huang M, Meuter ML, Challagalla G (2016). Getting smart. J Service Res.

[5] Larivière B, Bowen D, Andreassen TW, Kunz W, Sirianni NJ, Voss C, Wünderlich NV, De Keyser A (2017). “Service Encounter 2.0”: An investigation into the roles of technology, employees and customers. J Bus Res.

[6] Robinson S, Orsingher C, Alkire L, De Keyser A, Giebelhausen M, Papamichail KN, Shams P, Temerak MS (2020). Frontline encounters of the AI kind: An evolved service encounter framework. J Bus Res.

[7] Chi OH, Denton G, Gursoy D (2020). Artificially intelligent device use in service delivery: a systematic review, synthesis, and research agenda. J Hosp Mark Manag.

[8] Huang M, Rust RT (2020). A strategic framework for artificial intelligence in marketing. J Acad Mark Sci.

[9] De Keyser A, Köcher S, Alkire (née Nasr) L, Verbeeck C, Kandampully J (2019). Frontline service technology infusion: conceptual archetypes and future research directions. J Serv Manag.

[10] Gursoy D, Chi OH, Lu L, Nunkoo R (2019). Consumers acceptance of artificially intelligent (AI) device use in service delivery. Int J Inf Manag.

[11] López-Robles J, Otegi-Olaso J, Porto Gómez I, Cobo M (2019). 30 years of intelligence models in management and business: a bibliometric review. Int J Inf Manag.

[12] Coombs C, Hislop D, Taneva SK, Barnard S (2020). The strategic impacts of intelligent automation for knowledge and service work: an interdisciplinary review. J Strat Inf Syst.

[13] Khanna S (2013). Artificial intelligence in health – the three big challenges. Australas Med J.

[14] Dreyer K, Allen B (2018). Artificial intelligence in health care: brave new world or golden opportunity?. J Am Coll Radiol.

[15] Houssami N, Turner RM, Morrow M (2017). Meta-analysis of pre-operative magnetic resonance imaging (MRI) and surgical treatment for breast cancer. Breast Cancer Res Treat.

[16] Fraser KC, Meltzer JA, Rudzicz F (2015). Linguistic features identify Alzheimer’s disease in narrative speech. J Alzheimers Dis.

[17] Esteva A, Kuprel B, Novoa RA, Ko J, Swetter SM, Blau HM, Thrun S (2017). Dermatologist-level classification of skin cancer with deep neural networks. Nature.

[18] Hannun AY, Rajpurkar P, Haghpanahi M, Tison GH, Bourn C, Turakhia MP, Ng AY (2019). Cardiologist-level arrhythmia detection and classification in ambulatory electrocardiograms using a deep neural network. Nat Med.

[19] Wilson J, Daugherty PR (2018). Collaborative intelligence: humans and AI are joining forces. Harvard Business Review.

[20] Laï MC, Brian M, Mamzer M (2020). Perceptions of artificial intelligence in healthcare: findings from a qualitative survey study among actors in France. J Transl Med.

[21] Romero-Brufau S, Wyatt KD, Boyum P, Mickelson M, Moore M, Cognetta-Rieke C (2020). A lesson in implementation: a pre-post study of providers' experience with artificial intelligence-based clinical decision support. Int J Med Inform.

[22] Pinto Dos Santos D, Giese D, Brodehl S, Chon SH, Staab W, Kleinert R, Maintz D, Baeßler B (2019). Medical students' attitude towards artificial intelligence: a multicentre survey. Eur Radiol.

[23] Gong B, Nugent JP, Guest W, Parker W, Chang PJ, Khosa F, Nicolaou S (2019). Influence of artificial intelligence on Canadian medical students' preference for radiology specialty: a national survey study. Acad Radiol.

[24] European Society of Radiology (ESR) (2019). Impact of artificial intelligence on radiology: a EuroAIM survey among members of the European Society of Radiology. Insight Imag.

[25] Broadbent E, Tamagawa R, Patience A, Knock B, Kerse N, Day K, MacDonald BA (2012). Attitudes towards health-care robots in a retirement village. Australas J Ageing.

[26] Patel BN, Rosenberg L, Willcox G, Baltaxe D, Lyons M, Irvin J, Rajpurkar P, Amrhein T, Gupta R, Halabi S, Langlotz C, Lo E, Mammarappallil J, Mariano AJ, Riley G, Seekins J, Shen L, Zucker E, Lungren MP (2019). Human–machine partnership with artificial intelligence for chest radiograph diagnosis. NPJ Digit Med.

[27] Turja T, Aaltonen I, Taipale S, Oksanen A (2020). Robot acceptance model for care (RAM-care): A principled approach to the intention to use care robots. Inf Manag.

[28] Esmaeilzadeh P (2020). Use of AI-based tools for healthcare purposes: a survey study from consumers' perspectives. BMC Med Inform Decis Mak.

[29] Lee H, Piao M, Lee J, Byun A, Kim J (2020). The purpose of bedside robots: exploring the needs of inpatients and healthcare professionals. Comput Inform Nurs.

[30] Alami H, Rivard L, Lehoux P, Hoffman SJ, Cadeddu SBM, Savoldelli M, Samri MA, Ag Ahmed MA, Fleet R, Fortin J (2020). Artificial intelligence in health care: laying the foundation for responsible, sustainable, and inclusive innovation in low- and middle-income countries. Global Health.

[31] Zhang B, Dafoe A (2019). Artificial intelligence: American attitudes and trends. SSRN J.

[32] Esteva A, Robicquet A, Ramsundar B, Kuleshov V, DePristo M, Chou K, Cui C, Corrado G, Thrun S, Dean J (2019). A guide to deep learning in healthcare. Nat Med.

[33] Hermes S, Riasanow T, Clemons EK, Böhm M, Krcmar H (2020). The digital transformation of the healthcare industry: exploring the rise of emerging platform ecosystems and their influence on the role of patients. Bus Res.

[34] Holman H, Lorig K (2000). Patients as partners in managing chronic disease. Partnership is a prerequisite for effective and efficient health care. BMJ.

[35] Grichnik KP, Ferrante FM (1991). The difference between acute and chronic pain. Mt Sinai J Med.

[36] (2020). About chronic diseases. Centers for Disease Control and Prevention.

[37] Xu J (2018). Overtrust of robots in high-risk scenarios.

[38] Wu Y, Cristancho-Lacroix V, Fassert C, Faucounau V, de Rotrou J, Rigaud A (2016). The attitudes and perceptions of older adults with mild cognitive impairment toward an assistive robot. J Appl Gerontol.

[39] Bauchat JR, Seropian M, Jeffries PR (2016). Communication and empathy in the patient-centered care model—why simulation-based training is not optional. Clin Simul Nurs.

[40] Spiro H (2009). Commentary: the practice of empathy. Acad Med.

[41] Charon R (2001). The patient-physician relationship. Narrative medicine: a model for empathy, reflection, profession, and trust. JAMA.

[42] Kerasidou A (2020). Artificial intelligence and the ongoing need for empathy, compassion and trust in healthcare. Bull World Health Organ.

[43] Heinze K, Suwanabol PA, Vitous CA, Abrahamse P, Gibson K, Lansing B, Mody L (2020). A survey of patient perspectives on approach to health care: focus on physician competency and compassion. J Patient Exp.

[44] Singh P, King-Shier K, Sinclair S (2020). South Asian patients' perceptions and experiences of compassion in healthcare. Ethn Health.

[45] Kelley JM, Kraft-Todd G, Schapira L, Kossowsky J, Riess H (2014). The influence of the patient-clinician relationship on healthcare outcomes: a systematic review and meta-analysis of randomized controlled trials. PLoS One.

[46] Lu L, Cai R, Gursoy D (2019). Developing and validating a service robot integration willingness scale. Int J Hospit Manag.

[47] Vayena E, Blasimme A, Cohen IG (2018). Machine learning in medicine: Addressing ethical challenges. PLoS Med.

[48] Reddy S, Allan S, Coghlan S, Cooper P (2020). A governance model for the application of AI in health care. J Am Med Inform Assoc.

[49] Miller DD, Brown EW (2018). Artificial intelligence in medical practice: the question to the answer?. Am J Med.

[50] LaRosa E, Danks D (2018). Impacts on trust of healthcare AI.

[51] Bryson J, Winfield A (2017). Standardizing ethical design for artificial intelligence and autonomous systems. Computer.

[52] Gupta RK, Kumari R (2017). Artificial intelligence in public health: opportunities and challenges. JK Sci.

[53] Tang A, Tam R, Cadrin-Chênevert A, Guest W, Chong J, Barfett J, Chepelev L, Cairns R, Mitchell JR, Cicero MD, Poudrette MG, Jaremko JL, Reinhold C, Gallix B, Gray B, Geis R, Canadian Association of Radiologists (CAR) Artificial Intelligence Working Group (2018). Canadian Association of Radiologists White Paper on Artificial Intelligence in Radiology. Can Assoc Radiol J.

[54] Reddy S, Fox J, Purohit MP (2019). Artificial intelligence-enabled healthcare delivery. J R Soc Med.

[55] Luxton D (2019). Should Watson be consulted for a second opinion?. AMA J Ethics.

[56] Vance A, Elie-Dit-Cosaque C, Straub DW (2014). Examining trust in information technology artifacts: the effects of system quality and culture. J Manag Inf Syst.

[57] Whittlestone J, Nyrup R, Alexandrova A, Dihal K, Cave S (2019). Ethical and societal implications of algorithms, data, and artificial intelligence: a roadmap for research. Nuffield Foundation.

[58] Sun TQ, Medaglia R (2019). Mapping the challenges of artificial intelligence in the public sector: evidence from public healthcare. Gov Inf Quart.

[59] Lee J, Kim KJ, Lee S, Shin D (2015). Can autonomous vehicles be safe and trustworthy? Effects of appearance and autonomy of unmanned driving systems. Int J Hum-Comput Interact.

[60] Longoni C, Bonezzi A, Morewedge CK (2019). Resistance to medical artificial intelligence. J Consum Res.

[61] Hengstler M, Enkel E, Duelli S (2016). Applied artificial intelligence and trust—The case of autonomous vehicles and medical assistance devices. Technol Forecast Soc Change.

[62] He J, Baxter SL, Xu J, Xu J, Zhou X, Zhang K (2019). The practical implementation of artificial intelligence technologies in medicine. Nat Med.

[63] Mitchell M (2019). Artificial intelligence hits the barrier of meaning. Information.

[64] Angwin J, Larson J, Mattu S, Kirchner L (2016). Machine bias. ProPublica.

[65] Edwards SD (2018). The HeartMath coherence model: implications and challenges for artificial intelligence and robotics. AI Soc.

[66] Dwivedi YK, Hughes L, Ismagilova E, Aarts G, Coombs C, Crick T, Duan Y, Dwivedi R, Edwards J, Eirug A, Galanos V, Ilavarasan PV, Janssen M, Jones P, Kar AK, Kizgin H, Kronemann B, Lal B, Lucini B, Medaglia R, Le Meunier-FitzHugh K, Le Meunier-FitzHugh LC, Misra S, Mogaji E, Sharma SK, Singh JB, Raghavan V, Raman R, Rana NP, Samothrakis S, Spencer J, Tamilmani K, Tubadji A, Walton P, Williams MD (2021). Artificial intelligence (AI): multidisciplinary perspectives on emerging challenges, opportunities, and agenda for research, practice and policy. Int J Inf Manag.

[67] Stuart R, Norvig P (2016). Artificial intelligence-a modern approach 3rd edition.

[68] Kirkpatrick K (2017). It's not the algorithm, it's the data. Commun ACM.

[69] Noble SU (2021). Algorithms of oppression: how search engines reinforce racism NYU Press, 2018. 256 pp. Science.

[70] Cath C (2018). Governing artificial intelligence: ethical, legal and technical opportunities and challenges. Philos Trans A Math Phys Eng Sci.

[71] Esmaeilzadeh P (2019). The effects of public concern for information privacy on the adoption of Health Information Exchanges (HIEs) by healthcare entities. Health Commun.

[72] Dawson D, Schleiger E, Horton J, McLaughlin J, Robinson C, Quezada G, Scowcroft J, Hajkowicz S (2019). Artificial intelligence: Australia's ethics framework. Analysis and Policy Observatory.

[73] Zandi D, Reis A, Vayena E, Goodman K (2019). New ethical challenges of digital technologies, machine learning and artificial intelligence in public health: a call for papers. Bull World Health Organ.

[74] Beregi J, Zins M, Masson J, Cart P, Bartoli J, Silberman B, Boudghene F, Meder J, Conseil National Professionnel de la Radiologie et Imagerie Médicale (2018). Radiology and artificial intelligence: An opportunity for our specialty. Diagn Interv Imaging.

[75] Tizhoosh H, Pantanowitz L (2018). Artificial intelligence and digital pathology: challenges and opportunities. J Pathol Inform.

[76] Oakden-Rayner L (2019). Reply to 'Man against machine: diagnostic performance of a deep learning convolutional neural network for dermoscopic melanoma recognition in comparison to 58 dermatologists' by Haenssle et al. Ann Oncol.

[77] Sohn K, Kwon O (2020). Technology acceptance theories and factors influencing artificial Intelligence-based intelligent products. Telemat Inform.

[78] Jiang F, Jiang Y, Zhi H, Dong Y, Li H, Ma S, Wang Y, Dong Q, Shen H, Wang Y (2017). Artificial intelligence in healthcare: past, present and future. Stroke Vasc Neurol.

[79] Xu N, Wang K (2019). Adopting robot lawyer? The extending artificial intelligence robot lawyer technology acceptance model for legal industry by an exploratory study. J Manag Organiz.

[80] Sánchez-Prieto JC, Cruz-Benito J, Therón R, García-Peñalvo F (2020). Assessed by machines: development of a TAM-based tool to measure AI-based assessment acceptance among students. Int J Interact Multimed Artif Intell.

[81] Zhao X, Xia Q, Huang W (2020). Impact of technostress on productivity from the theoretical perspective of appraisal and coping processes. Inf Manag.

[82] Marakanon L, Panjakajornsak V (2017). Perceived quality, perceived risk and customer trust affecting customer loyalty of environmentally friendly electronics products. Kasetsart J Soc Sci.

[83] Zhang X, Liu S, Chen X, Wang L, Gao B, Zhu Q (2018). Health information privacy concerns, antecedents, and information disclosure intention in online health communities. Inf Manag.

[84] Lo WLA, Lei D, Li L, Huang DF, Tong K (2018). The perceived benefits of an artificial intelligence-embedded mobile app implementing evidence-based guidelines for the self-management of chronic neck and back pain: observational study. JMIR Mhealth Uhealth.

[85] Cohen J (1992). A power primer. Psychol Bull.

[86] Paolacci G, Chandler J (2014). Inside the Turk. Curr Dir Psychol Sci.

[87] Hair JF, Ringle CM, Sarstedt M (2014). PLS-SEM: indeed a silver bullet. J Mark Theory Pract.

[88] Froomkin AM, Kerr IR, Pineau J (2019). When AIs outperform doctors: the dangers of a tort-induced over-reliance on machine learning and what (not) to do about it. Ariz Law Rev.

[89] Xu J, Yang P, Xue S, Sharma B, Sanchez-Martin M, Wang F, Beaty KA, Dehan E, Parikh B (2019). Translating cancer genomics into precision medicine with artificial intelligence: applications, challenges and future perspectives. Hum Genet.

[90] Lee S, Lee N, Sah YJ (2019). Perceiving a mind in a chatbot: effect of mind perception and social cues on co-presence, closeness, and intention to use. Int J Hum-Comput Interact.

[91] Sharkey A, Sharkey N (2010). Granny and the robots: ethical issues in robot care for the elderly. Ethics Inf Technol.

[92] Duan Y, Edwards JS, Dwivedi YK (2019). Artificial intelligence for decision making in the era of Big Data – evolution, challenges and research agenda. Int J Inf Manag.

[93] Pesapane F, Volonté C, Codari M, Sardanelli F (2018). Artificial intelligence as a medical device in radiology: ethical and regulatory issues in Europe and the United States. Insight Imag.

[94] Waring J, Lindvall C, Umeton R (2020). Automated machine learning: Review of the state-of-the-art and opportunities for healthcare. Artif Intell Med.

[95] Tran V, Riveros C, Ravaud P (2019). Patients' views of wearable devices and AI in healthcare: findings from the ComPaRe e-cohort. NPJ Digit Med.

[96] Fiske A, Henningsen P, Buyx A (2019). Your robot therapist will see you now: ethical implications of embodied artificial intelligence in psychiatry, psychology, and psychotherapy. J Med Internet Res.

[97] Schiff D, Borenstein J (2019). How should clinicians communicate with patients about the roles of artificially intelligent team members?. AMA J Ethics.

