# Supplementary material for: Patients’ Perceptions Toward Human–Artificial Intelligence Interaction in Health Care: Experimental Study
Source: J Med Internet Res. 2021 Nov 25;23(11):e25856. doi: 10.2196/25856 (PMC8663518; doi:10.2196/25856)
Supplement: Multimedia Appendix 1 [file jmir_v23i11e25856_app1.docx]

**Appendix 1: Experiments and Scenarios**

**Note**: It should be mentioned that participants of this study were actually suffering from either a chronic or acute disease. First, individuals entered their signs and symptoms to highlight what diseases they are suffering from. A filtering question was included at the beginning of the survey to categorize patients into chronic or acute groups (based on the data entered). Then, each group was randomly assigned to a hypothetical clinical encounter (one of the following six experiments).

**Experiment 1-1:**

Dear participants,

Please read the provided definition of AI and case scenario first, and then answer the following questions accordingly.

**Artificial Intelligence (AI) definition:**

Artificial Intelligence (AI) simply means that a machine (e.g., a computerized system) is able to perform a task that needs physical ability or mental intelligence and previously could only be done by human beings. AI can perform these tasks or make decisions **without explicit human instructions.**For example, AI could be a computer application that is good at performing a single task, such as playing chess or poker. Thus, AI could perform a task better than a human.

**Scenario:**

**AI clinical applications for health care purposes:**

AI embedded in Mobile health devices or health Apps could help patients monitor their health status, check their health care information, and manage their chronic illnesses. These AI clinical applications use algorithms to learn from the past by analyzing past medical histories of patients with the same health conditions, recognizing patterns in clinical data, predicting possible health issues, and suggesting some treatment choices, diagnostic options, prescription advice, and care planning. These devices could reduce frequent patient-physician encounters and avoid unnecessary hospitalizations.

- **Health condition: acute disease:**

Based on the data entered, you believe that you are suffering from an acute health condition. In this scenario, we only focus on **acute** health conditions (**NOT chronic diseases**). **Acute** illnesses generally develop suddenly, are severe and sudden in onset (the initial phase of a disease or condition, in which symptoms first become apparent),  and last a short time, often only a few days or weeks, and can be cured.

**Acute** diseases come on rapidly and are accompanied by distinct symptoms that require urgent or short-term care and get better once they are treated. For example, a broken bone that might result from a fall must be treated by a doctor and will heal in time. Sometimes, an acute illness, such as the common cold, will just go away on its own. Most people with acute illnesses will soon recover.

Some examples:  Common cold and Flu.

- **The healthcare service encounter for this scenario:**

Now, you have the option of using an AI clinical application. First, you enter your signs, symptoms, and critical health complaints into the AI clinical application. Your health information will be recorded in a huge database. Then, the AI system analyzes your health data and compares them to the learned patterns (for example, the list of diseases and medicines), and draws some clinical conclusions. Finally, based on the pattern found, it creates a report including:

-  Some diagnostic options

-  Some treatment choices

-  Prescription advice (e.g., dose, frequency, and name of medications you need to take)

-  Care planning (e.g., resting at home, taking suggested medicines for a specific period, or visiting a professional immediately)

In summary, AI clinical applications are able to analyze clinical data and make medical decisions for patients **without direct physician interactions**. So, we can consider this scenario as **using AI clinical applications without physician interactions for treating acute diseases.**

 Please answer the following questions considering the mentioned case scenario (AI clinical applications for acute diseases). Thank you very much for your time.

**Experiment 1-2:**

Dear participants,

Please read the provided definition of AI and case scenario first, and then answer the following questions accordingly.

**Artificial Intelligence (AI) definition:**

Artificial Intelligence (AI) simply means that a machine (e.g., a computerized system) is able to perform a task that needs physical ability or mental intelligence and previously could only be done by human beings. AI can perform these tasks or make decisions **without explicit human instructions**.  For example, AI could be a computer application that is good at performing a single task, such as playing chess or poker. Thus, AI could perform a task better than a human.

**Scenario:**

**AI clinical applications for health care purposes:**

AI embedded in Mobile health devices or health Apps could help patients monitor their health status, check their health care information, and manage their chronic illnesses. These AI clinical applications use algorithms to learn from the past by analyzing past medical histories of patients with the same health conditions, recognizing patterns in clinical data, predicting possible health issues, and suggesting some treatment choices, diagnostic options, prescription advice, and care planning. These devices could reduce frequent patient-physician encounters and avoid unnecessary hospitalizations.

- **Health condition: Chronic disease:**

Based on the data entered, you believe that you are suffering from a chronic health condition. In this scenario, we only focus on chronic health conditions (**NOT acute diseases**). A chronic condition is a human health condition or disease that is persistent or otherwise long-lasting in its effects or a disease that comes with time.

**Chronic** conditions develop slowly and may worsen over an extended period of time—months to years.**Chronic** conditions are slower to develop, may progress over time, and may have any number of warning signs or no signs at all. Common chronic conditions are arthritis, Alzheimer's disease, diabetes, heart disease, depression, high blood pressure, high cholesterol, and chronic kidney disease. Unlike acute conditions, chronic health conditions cannot be cured—only controlled.

- **The healthcare service encounter for this scenario:**

Now, you have the option of using an AI clinical application. First, you enter your signs, symptoms, and critical health complaints into the AI clinical application. Your health information will be recorded in a huge database. Then, the AI system analyzes your health data and compares them to the learned patterns (for example, the list of diseases and medicines), and draws some clinical conclusions. Finally, based on the pattern found, it creates a report including:

-  Some diagnostic options

-  Some treatment choices

-  Prescription advice (e.g., dose, frequency, and name of medications you need to take)

-  Care planning (e.g., resting at home, taking suggested medicines for a specific period, or visiting a professional immediately)

In summary, AI clinical applications are able to analyze clinical data and make medical decisions for patients **without direct physician interactions**. So, we can consider this scenario as **using AI clinical applications without physician interactions for controlling chronic diseases.**

 Please answer the following questions considering the mentioned case scenario (AI clinical applications for chronic diseases). Thank you very much for your time.

The survey used for experiments 1-2 and 1-2:

**1. Perceived Performance Risks**

I am concerned that the mechanisms used by AI clinical applications may lead to inaccurate predictions

I am concerned that the mechanisms used by AI clinical applications may result in medical errors

I am concerned that treatments provided by AI clinical applications may be incomplete

I am concerned that the predictive models of AI clinical applications may malfunction

I am concerned that the medical decisions made by AI clinical applications may be inadequate

**2. Perceived Social Biases**

I am concerned that the AI clinical applications may overestimate or underestimate health risks in a certain patient population (e.g., people with insufficient data in AI datasets)

I am concerned that data used in the AI clinical applications may lead to societal discrimination to a certain patient group (e.g., minority groups)

I am concerned that AI clinical applications used in healthcare may be unfair to a certain group of population (e.g., people with poor access to health care)

I am concerned that AI clinical applications could lead to morally flawed practices in health care

Overall, I am concerned that the possibility of biases by AI clinical applications to certain groups of the population is high

**3. Perceived Privacy Concerns**

I think using AI clinical applications helps health care entities collect too much personal information from me

I think in this case, I am concerned that health care entities use my health information for other purposes without my knowledge and authorization

In this case, I am concerned that my health information will be shared with other entities without my explicit consent

In this case, I am concerned that unauthorized people will have access to my health information

In this case, I am concerned about the privacy of my health information through using AI clinical applications

In this case, I am concerned my health information would be sold to others without my permission

**4. Perceived Trust**

**I trust in the** AI clinical applications **used for healthcare delivery**

**I trust in the** AI clinical applications **used in the healthcare**

**I trust in** AI clinical applications' **predictive and diagnostic ability for treatment purposes**

**I trust in the accuracy and predictive powers of current AI algorithmic models used in the medical context**

**I trust that** AI clinical applications **can adapt to specific and unforeseen medical situations.**

**5. Perceived Communication Barriers**

**I am concerned that** AI clinical applications **may eliminate the contact between healthcare professionals and patients**

**I am concerned that** AI clinical applications **may reduce conversation between physicians and patients**

**I am concerned that** AI clinical applications **may decrease human- aspects of relations in the medical contexts**

**I am concerned that by using** AI clinical applications**, I may lose face-to-face cues and personal interactions with physicians**

**I am concerned that by using** AI clinical applications**, I may be in a more passive position for making medical decisions**

**6. Perceived Transparency of Regulatory Standards**

**I am concerned that special policies and guidelines for** AI clinical applications **are not transparent yet**

**I am concerned that the safety and efficacy of** AI clinical applications **are not regulated clearly**

**I am concerned that regulatory standards to assess AI algorithmic safety are yet to be formalized**

**I am concerned that an appropriate regulatory and accreditation system regarding** AI clinical applications **is not in place yet**

**I am concerned about the lack of clear guidelines to monitor the performance of** AI clinical applications **in the medical context**

**7. Perceived Liability Issues**

**I am concerned because it is not clear who is responsible when errors result from the use of** AI clinical applications

**I am concerned about the liability of using** AI clinical applications **for my healthcare**

**I am concerned because it is not clear who becomes responsible if** AI clinical applications **offer wrong recommendations**

**I am concerned because it is unclear where the lines of responsibility begin or end when** AI clinical applications **guide clinical care**

**I am concerned because it is not clear who is responsible if appropriate AI-recommended treatment options are mistakenly dismissed**

**Overall, I am concerned that the use of** AI clinical applications **for clinical purposes increases my liability**

**8. Perceived Benefits**

**I believe** AI clinical applications **can improve diagnostics**

**I think** AI clinical applications **can enhance prognosis**

**I believe** AI clinical applications **can advance patient management systems**

**I believe** AI clinical applications **can suggest accurate care planning**

**I think** AI clinical applications **can recommend reliable treatment options**

**I think** AI clinical applications **can reduce healthcare costs**

**Overall, I think** AI clinical applications **can boost healthcare outcomes**

**9. Intention to Use**

**I agree to use** AI clinical applications **for clinical purposes**

**Using** AI clinical applications **for healthcare purposes is something I would consider**

**I would like to use** AI clinical applications **to manage my healthcare**

**In the future, I am willing to use** AI clinical applications **for diagnostics and treatments**

**I am very likely to use recommendations provided by** AI clinical applications **for care planning**

**Personal Innovativeness in Domain of Information Technology (IT)**

**If I heard about new information technology (IT), I would look for ways to experiment with it**

**Among my peers, I am usually the first to try out new information technologies**

**In general, I am not hesitant to try out new information technologies**

**I like to experiment with new information technologies**

Gender

**Male**

**Female**

Age

**Under 20**

**20-29**

**30-39**

**40-49**

**50-59**

**60 or older**

Annual household income

**Less than $25,000**

**$25,000-$49,999**

**$50,000-$74,999**

**$75,000-$99,999**

**$100,000-$150,000**

**More than $150,000**

Highest level of education

**Less than high school**

**High school graduate**

**Some college**

**2-year degree**

**Bachelor's degree**

**Master's degree**

**Doctorate**

Employment

**Employed full time**

**Employed part-time**

**Unemployed**

**Retired**

**Student**

Race/Ethnicity

**White**

**African American**

**Asian**

**Hispanic**

**Mixed**

**Other**

Have you ever used any AI clinical applications for any reason except for healthcare? (Such as AI embedded in smart devices for any purposes such as financial decision-making)

**Yes**

**No**

Generally, how familiar are you with an AI application (used for any purposes except for healthcare)?

**Extremely familiar**

**Very familiar**

**Moderately familiar**

**Slightly familiar**

**Not familiar at all**

Have you ever used any AI clinical applications?

**Yes**

**No**

How familiar are you with these AI clinical applications used for clinical and treatment purposes?

**Extremely familiar**

**Very familiar**

**Moderately familiar**

**Slightly familiar**

**Not familiar at all**

Overall, do you think your health information is .....?

**Sensitive**

**Non-sensitive**

**I have no idea**

How do you generally rate your computer skills?

**Excellent**

**Good**

**Average**

**Poor**

**Terrible**

How do you rate your technical knowledge about AI technology?

**Excellent**

**Good**

**Average**

**Poor**

**Terrible**

How do you rate your health literacy?

**Excellent**

**Good**

**Average**

**Poor**

**Terrible**

**Experiment 2-1:**

Dear participants,

Please read the provided definition and case scenario first, and then answer the following questions accordingly.

**Artificial Intelligence (AI) definition:**

Artificial Intelligence (AI) simply means that a machine (e.g., a computerized system) is able to perform a task that needs physical ability or mental intelligence and previously could only be done by human beings. AI can perform these tasks or make decisions without explicit human instructions.  For example, AI could be a computer application that is good at performing a single task, such as playing chess or poker. Thus, AI could perform a task better than a human.

**Scenario:**

**AI clinical applications for health care purposes:**

AI embedded in Mobile health devices or health Apps could help patients monitor their health status, check their health care information, and manage their chronic illnesses. These AI clinical applications use algorithms to learn from the past by analyzing past medical histories of patients with the same health conditions, recognizing patterns in clinical data, predicting possible health issues, and suggesting some treatment choices, diagnostic options, prescription advice, and care planning. These devices could reduce frequent patient-physician encounters and avoid unnecessary hospitalizations.

- **Health condition: acute disease:**

Based on the data entered, you believe that you are suffering from an acute health condition. In this scenario, we only focus on **acute** health conditions (**NOT chronic diseases**). **Acute** illnesses generally develop suddenly, are severe and sudden in onset (the initial phase of a disease or condition, in which symptoms first become apparent),  and last a short time, often only a few days or weeks, and can be cured.

**Acute** diseases come on rapidly and are accompanied by distinct symptoms that require urgent or short-term care and get better once they are treated. For example, a broken bone that might result from a fall must be treated by a doctor and will heal in time. Sometimes, an acute illness, such as the common cold, will just go away on its own. Most people with acute illnesses will soon recover.

Some examples:  Common cold and Flu.

- **The healthcare service encounter for this scenario:**

Now, you have the option of using an AI clinical application that is **monitored and controlled by your physician**. First, you enter your signs, symptoms, and critical health complaints into the AI clinical application. Your health information will be recorded in a huge database. Then, the AI system analyzes your health data and compares them to the learned patterns (for example, the list of diseases and medicines), and draws some clinical conclusions. Finally, based on the pattern found, it creates a report including:

-  Some diagnostic options

-  Some treatment choices

-  Prescription advice (e.g., dose, frequency, and name of medications you need to take)

-  Care planning (e.g., resting at home, taking suggested medicines for a specific period, or visiting a specialist immediately)

 Then, your **doctor will check the results and recommendations** generated by the AI clinical application, discard some of the recommendations based on his/her experience and expertise, and make the final decision about your treatment choices, prescription options, and care planning.

In summary, AI clinical applications are able to analyze clinical data and help physicians make medical decisions for patients. Thus, in this case, **AI clinical applications are used with the direct supervision of physicians**. So, we can consider this scenario as **AI-physician interactions (Interactions between physicians and AI clinical applications) for treating acute diseases**.

 Please answer the following questions considering the mentioned case scenario. Thank you very much for your time.

**Experiment 2-2:**

Dear participants,

Please read the provided definition and case scenario first, and then answer the following questions accordingly.

**Artificial Intelligence (AI) definition:**

Artificial Intelligence (AI) simply means that a machine (e.g., a computerized system) is able to perform a task that needs physical ability or mental intelligence and previously could only be done by human beings. AI can perform these tasks or make decisions without explicit human instructions.  For example, AI could be a computer application that is good at performing a single task, such as playing chess or poker. Thus, AI could perform a task better than a human.

**Scenario:**

**AI clinical applications for health care purposes:**

AI embedded in Mobile health devices or health Apps could help patients monitor their health status, check their health care information, and manage their chronic illnesses. These AI clinical applications use algorithms to learn from the past by analyzing past medical histories of patients with the same health conditions, recognizing patterns in clinical data, predicting possible health issues, and suggesting some treatment choices, diagnostic options, prescription advice, and care planning. These devices could reduce frequent patient-physician encounters and avoid unnecessary hospitalizations.

- **Health condition: Chronic disease:**

Based on the data entered, you believe that you are suffering from a chronic health condition. In this scenario, we only focus on chronic health conditions (**NOT acute diseases**). A chronic condition is a human health condition or disease that is persistent or otherwise long-lasting in its effects or a disease that comes with time.

**Chronic** conditions develop slowly and may worsen over an extended period of time—months to years.**Chronic** conditions are slower to develop, may progress over time, and may have any number of warning signs or no signs at all. Common chronic conditions are arthritis, Alzheimer's disease, diabetes, heart disease, depression, high blood pressure, high cholesterol, and chronic kidney disease. Unlike acute conditions, chronic health conditions cannot be cured—only controlled.

- **The healthcare service encounter for this scenario:**

Now, you have the option of using an AI clinical application that is **monitored and controlled by your physician**. First, you enter your signs, symptoms, and critical health complaints into the AI clinical application. Your health information will be recorded in a huge database. Then, the AI system analyzes your health data and compares them to the learned patterns (for example, the list of diseases and medicines), and draws some clinical conclusions. Finally, based on the pattern found, it creates a report including:

-  Some diagnostic options

-  Some treatment choices

-  Prescription advice (e.g., dose, frequency, and name of medications you need to take)

-  Care planning (e.g., resting at home, taking suggested medicines for a specific period or visiting a specialist immediately)

Then, your **doctor will check the results and recommendations** generated by the AI clinical application, discard some of the recommendations based on his/her experience and expertise, and make the final decision about your treatment choices, prescription options, and care planning.

In summary, AI clinical applications are able to analyze clinical data and help physicians make medical decisions for patients. Thus, in this case, **AI clinical applications are used with the direct supervision of physicians**. So, we can consider this scenario as **AI-physician interactions (Interactions between physicians and AI clinical applications) for controlling chronic diseases**.

 Please answer the following questions considering the mentioned case scenario. Thank you very much for your time.

The survey used for experiments 2-1 and 2-2:

**1. Perceived Performance Risks**

I am concerned that AI-physician interactions may lead to inaccurate predictions

I am concerned that the AI-physician interactions may result in medical errors

I am concerned that treatments provided by AI-physician interactions may be incomplete

I am concerned that the AI-physician interactions may not work in a proper way

I am concerned that the medical decisions made through AI-physician interactions may be inadequate

**2. Perceived Social Biases**

I am concerned that the AI-physician interactions may overestimate or underestimate health risks in a certain patient population (e.g., specific race)

I am concerned that AI-physician interactions may lead to societal discrimination to a certain patient group (e.g., minority groups)

I am concerned that AI-physician interactions may be unfair to a certain group of population (e.g., people with poor access to health care)

I am concerned that AI-physician interactions could give rise to morally flawed practices in health care

Overall, I am concerned that the possibility of biases by AI-physician interactions to certain groups of the population is high

**3. Perceived Privacy Concerns**

In this case, health care entities could collect too much personal information from me

I think in this case, I am concerned that health care entities use my health information for other purposes without my knowledge and authorization

In this case, I am concerned that my health information will be shared with other entities without my explicit consent

In this case, I am concerned that unauthorized people will have access to my health information

In this case, I am concerned about the privacy of my health information through using AI clinical applications

In this case, I am concerned my health information would be sold to others without my permission

**4. Perceived Trust**

**I trust in AI-physician interactions for healthcare delivery**

**I trust in AI-physician interactions in the examination process**

**I trust in AI-physician interactions' predictive and diagnostic ability for treatment purposes**

**I trust in the interaction between physicians and** AI clinical applications **during consultations**

**I trust that the interaction between physicians and** AI clinical applications **can adapt to specific and unforeseen medical situations.**

**5. Perceived Communication Barriers**

**I am concerned that AI-physician interactions may eliminate the contact between healthcare professionals and patients**

**I am concerned that AI-physician interactions may reduce conversation between physicians and patients**

**I am concerned that AI-physician interactions may decrease human- aspects of relations in the medical contexts**

**I am concerned that through the interaction between physicians and** AI clinical applications **, I may lose face-to-face cues and personal interactions with physicians**

**I am concerned that due to AI-physician interactions, I may be in a more passive position for making medical decisions**

**6. Perceived Transparency of Regulatory Standards**

**I am concerned that special policies and guidelines for AI-physician interactions are not transparent yet**

**I am concerned that the safety and efficacy of AI-physician interactions are not regulated clearly**

**I am concerned that regulatory standards to assess the safety of AI-physician interactions are yet to be formalized**

**I am concerned that an appropriate regulatory and accreditation system regarding AI-physician interactions is not in place yet**

**I am concerned about the lack of clear guidelines to monitor the performance of AI-physician interactions in the medical context**

**7. Perceived Liability Issues**

**I am concerned because it is not clear who is responsible when errors result from the use of** AI clinical applications **by physicians**

**I am concerned about the liability of using** AI clinical applications under **the control of physicians for my healthcare**

**I am concerned because it is not clear who becomes responsible if the interaction between physicians and** AI clinical applications **offer wrong recommendations**

**I am concerned because it is unclear where the lines of responsibility begin or end when AI-physician interactions guide clinical care**

**I am concerned because it is not clear who is responsible if appropriate AI-recommended treatment options are mistakenly dismissed by physicians**

**Overall, I am concerned that the use of AI-physician interactions for clinical purposes increases my liability**

**8. Perceived Benefits**

**I believe the interaction between physicians and** AI clinical applications **can improve diagnostics**

**I think the interaction between physicians and** AI clinical applications **can enhance prognosis**

**I believe the interaction between physicians and** AI clinical applications **can advance patient management systems**

**I believe the interaction between physicians and** AI clinical applications **can suggest accurate care planning**

**I think the interaction between physicians and** AI clinical applications **can recommend reliable treatment options**

**I think the interaction between physicians and** AI clinical applications **can reduce healthcare costs**

**Overall, I think the interaction between physicians and** AI clinical applications **can boost healthcare outcomes**

**9. Intention to Use**

**I agree to use AI-physician interactions for clinical purposes**

**Using AI-physician interactions for healthcare purposes is something I would consider**

**I would like to use AI-physician interactions to manage my healthcare**

**In the future, I am willing to use AI-physician interactions for diagnostics and treatments**

**I am very likely to use recommendations provided by AI-physician interactions for care planning**

**Personal Innovativeness in Domain of Information Technology (IT)**

**If I heard about new information technology (IT), I would look for ways to experiment with it**

**Among my peers, I am usually the first to try out new information technologies**

**In general, I am not hesitant to try out new information technologies**

**I like to experiment with new information technologies**

Gender

**Male**

**Female**

Age

**Under 20**

**20-29**

**30-39**

**40-49**

**50-59**

**60 or older**

Annual household income

**Less than $25,000**

**$25,000-$49,999**

**$50,000-$74,999**

**$75,000-$99,999**

**$100,000-$150,000**

**More than $150,000**

Highest level of education

**Less than high school**

**High school graduate**

**Some college**

**2-year degree**

**Bachelor's degree**

**Master's degree**

**Doctorate**

Employment

**Employed full time**

**Employed part-time**

**Unemployed**

**Retired**

**Student**

Race/Ethnicity

**White**

**African American**

**Asian**

**Hispanic**

**Mixed**

**Other**

Have you ever used any AI-enabled services or devices for any reason except for healthcare? (Such as AI embedded in smart devices for any purposes such as financial decision-making)

**Yes**

**No**

Generally, how familiar are you with an AI application (used for any purposes except for healthcare)?

**Extremely familiar**

**Very familiar**

**Moderately familiar**

**Slightly familiar**

**Not familiar at all**

Have you ever used any AI clinical applications?

**Yes**

**No**

How familiar are you with these AI clinical applications used for clinical purposes?

**Extremely familiar**

**Very familiar**

**Moderately familiar**

**Slightly familiar**

**Not familiar at all**

Overall, do you think your health information is .....?

**Sensitive**

**Non-sensitive**

**I have no idea**

How do you generally rate your computer skills?

**Excellent**

**Good**

**Average**

**Poor**

**Terrible**

How do you rate your technical knowledge about AI technology?

**Excellent**

**Good**

**Average**

**Poor**

**Terrible**

How do you rate your health literacy?

**Excellent**

**Good**

**Average**

**Poor**

**Terrible**

**Experiment 3-1:**

Dear participants,

Please read the provided definition and case scenario first, and then answer the following questions accordingly.

**Scenario:**

**Traditional in-person examinations:**

Based on the data entered, you believe that you are suffering from an acute health condition. In this scenario, we only focus on **acute** health conditions (**NOT chronic diseases**). **Acute** illnesses generally develop suddenly, are severe and sudden in onset (the initial phase of a disease or condition, in which symptoms first become apparent),  and last a short time, often only a few days or weeks, and can be cured.

**Acute** diseases come on rapidly and are accompanied by distinct symptoms that require urgent or short-term care and get better once they are treated. For example, a broken bone that might result from a fall must be treated by a doctor and will heal in time. Sometimes, an acute illness, such as the common cold, will just go away on its own. Most people with acute illnesses will soon recover.

Some examples:  Common cold and Flu.

- **The healthcare service encounter for this scenario:**

Now, you have the option of visiting your doctor. First, your doctor asks about your signs, symptoms, and critical health complaints through a conventional in-person examination. The doctor analyzes your health information. Then, based on his/her knowledge, expertise, and experience, he/she draws some clinical conclusions such as:

-  The best diagnostic options

-  The best treatment choices

-  Prescription advice (e.g., dose, frequency, and name of medications you need to take)

-  Care planning (e.g., resting at home, taking suggested medicines for a specific period, or visiting a specialist immediately)

In summary, in this case, through a face-to-face patient/physician encounter, your doctor is able to analyze clinical data and make final medical decisions for you. So, we can consider this scenario as **in-person examinations with physicians for treating acute diseases**.

 Please answer the following questions considering the mentioned case scenario. Thank you very much for your time.

**Experiment 3-2:**

Dear participants,

Please read the provided definition and case scenario first, and then answer the following questions accordingly.

**Scenario:**

**Traditional in-person examinations:**

- **Health condition: Chronic disease:**

Based on the data entered, you believe that you are suffering from a chronic health condition. In this scenario, we only focus on chronic health conditions (**NOT acute diseases**). A chronic condition is a human health condition or disease that is persistent or otherwise long-lasting in its effects or a disease that comes with time.

**Chronic** conditions develop slowly and may worsen over an extended period of time—months to years.**Chronic** conditions are slower to develop, may progress over time, and may have any number of warning signs or no signs at all. Common chronic conditions are arthritis, Alzheimer's disease, diabetes, heart disease, depression, high blood pressure, high cholesterol, and chronic kidney disease. Unlike acute conditions, chronic health conditions cannot be cured—only controlled.

- **The healthcare service encounter for this scenario:**

 Now, you have the option of visiting your doctor. First, your doctor asks about your signs, symptoms, and critical health complaints through a conventional in-person examination. The doctor analyzes your health information. Then, based on his/her knowledge, expertise, and experience, he/she draws some clinical conclusions such as:

-  The best diagnostic options

-  The best treatment choices

-  Prescription advice (e.g., dose, frequency, and name of medications you need to take)

-  Care planning (e.g., resting at home, taking suggested medicines for a specific period, or visiting a specialist immediately)

In summary, in this case, through a face-to-face patient/physician encounter, your doctor is able to analyze clinical data and make final medical decisions for you. So, we can consider this scenario as **in-person examinations with physicians for controlling chronic diseases**.

 Please answer the following questions considering the mentioned case scenario. Thank you very much for your time.

The survey used for experiments 3-1 and 3-2:

**1. Perceived Performance Risks**

I am concerned that physicians may make inaccurate predictions

I am concerned that physicians may cause medical errors

I am concerned that treatments provided by physicians may be incomplete

I am concerned that physicians may not practice in a proper way

I am concerned that the medical decisions made by physicians may be inadequate

**2. Perceived Social Biases**

I am concerned that physicians may overestimate or underestimate health risks in a certain patient population (e.g., a particular race)

I am concerned that physicians may create societal discrimination to a certain patient group (e.g., minority groups)

I am concerned that physicians may be unfair to a certain group of population (e.g., people with poor access to health care)

I am concerned that physicians could give rise to morally flawed practices in health care

Overall, I am concerned that the possibility of biases by physicians to certain groups of the population is high

**3. Perceived Privacy Concerns**

In this case, health care entities could collect too much personal information from me

I think in this case, I am concerned that health care entities use my health information for other purposes without my knowledge and authorization

In this case, I am concerned that my health information will be shared with other entities without my explicit consent

In this case, I am concerned that unauthorized people will have access to my health information

In this case, I am concerned about the privacy of my health information

In this case, I am concerned my health information would be sold to others without my permission

**4. Perceived Trust** I trust in the expertise of physicians for healthcare delivery

I trust in the experience of physicians in the examination process

I trust in physicians' predictive and diagnostic ability for treatment purposes

I trust in the knowledge of physicians during consultations

I trust that physicians can adapt to specific and unforeseen medical situations

**5. Perceived Communication Barriers**

I am concerned that the contact between physicians and patients is not effective during in-person examinations

I am concerned that the conversation between physicians and patients is not satisfactory during in-person examinations

I am concerned about human- aspects of relations in medical examinations

I am concerned about the face-to-face cues and personal interactions with physicians during in-person examinations

I am concerned that in the conventional examination, I may be in a more passive position for making medical decisions

**6. Perceived Transparency of Regulatory Standards**

I am concerned that special policies and guidelines for physician practices are not transparent yet

I am concerned that the safety and efficacy of physician practices are not regulated clearly

I am concerned that regulatory standards to assess the safety of physician practices are yet to be formalized

I am concerned that appropriate regulatory and accreditation system regarding physician practices is not in place yet

I am concerned about the lack of clear guidelines to monitor the performance of physicians in the medical context

**7. Perceived Liability Issues**

I am concerned because it is not clear who is responsible when errors occur as a result of physician practices

I am concerned about the direct liability of physicians during consultations

I am concerned because it is not clear who becomes responsible if physicians offer wrong recommendations

I am concerned because it is unclear where the lines of responsibility begin or end when physicians directly guide clinical care

I am concerned because it is not clear who is responsible if physicians mistakenly dismiss my appropriate treatment suggestions.

Overall, I am concerned that the use of in-person examinations increases my liability

**8. Perceived Benefits**

I believe in-person examinations can improve diagnostics

I think in-person examinations can enhance prognosis

I believe in-person examinations can advance patient management systems

I believe in-person examinations can generate accurate care planning

I think in-person examinations can produce reliable treatment options

I think in-person examinations can reduce healthcare costs

Overall, I think in-person examinations can boost healthcare outcomes

**9. Intention to Use**

I agree to use in-person examinations for clinical purposes

Using in-person examinations for healthcare purposes is something I would consider

I would like to use in-person examinations to manage my healthcare

In the future, I am willing to use in-person examinations for diagnostics and treatments

I am very likely to use recommendations provided by physicians for care planning

**Personal Innovativeness in Domain of Information Technology (IT)**

**If I heard about a new information technology (IT), I would look for ways to experiment with it**

**Among my peers, I am usually the first to try out new information technologies**

**In general, I am not hesitant to try out new information technologies**

**I like to experiment with new information technologies**

Gender

**Male**

**Female**

Age

**Under 20**

**20-29**

**30-39**

**40-49**

**50-59**

**60 or older**

Annual household income

**Less than $25,000**

**$25,000-$49,999**

**$50,000-$74,999**

**$75,000-$99,999**

**$100,000-$150,000**

**More than $150,000**

Highest level of education

**Less than high school**

**High school graduate**

**Some college**

**2 year degree**

**Bachelor's degree**

**Master's degree**

**Doctorate**

Employment

**Employed full time**

**Employed part time**

**Unemployed**

**Retired**

**Student**

Race/Ethnicity

**White**

**African American**

**Asian**

**Hispanic**

**Mixed**

**Other**

How familiar are you with your physicians?

**Extremely familiar**

**Very familiar**

**Moderately familiar**

**Slightly familiar**

**Not familiar at all**

Overall, do you think your health information is .....?

**Sensitive**

**Non-sensitive**

**I have no idea**

How do you generally rate your computer skills?

**Excellent**

**Good**

**Average**

**Poor**

**Terrible**

How do you rate your health literacy?

**Excellent**

**Good**

**Average**

**Poor**

**Terrible**
