# Supplementary material for: Patients’ Perceptions Toward Human–Artificial Intelligence Interaction in Health Care: Experimental Study
Source: J Med Internet Res. 2021 Nov 25;23(11):e25856. doi: 10.2196/25856 (PMC8663518; doi:10.2196/25856)
Supplement: Multimedia Appendix 2 [file jmir_v23i11e25856_app2.docx]

**Appendix 2:**

**Demographic information of the six scenarios:**

**1. Gender:**

|  |  |  | Scenario | | | | | | Total |
| --- | --- | --- | --- | --- | --- | --- | --- | --- | --- |
|  |  |  | 1-1 | 1-2 | 2-1 | 2-2 | 3-1 | 3-2 |  |
| Gender | Male | Count | 59 | 58 | 69 | 59 | 55 | 58 | 358 |
|  |  | % within Scenario | 56.2% | 55.8% | 61.1% | 57.3% | 52.4% | 55.8% | 56.5% |
|  | Female | Count | 46 | 46 | 44 | 44 | 50 | 46 | 276 |
|  |  | % within Scenario | 43.8% | 44.2% | 38.9% | 42.7% | 47.6% | 44.2% | 43.5% |
| Total | | Count | 105 | 104 | 113 | 103 | 105 | 104 | 634 |
|  |  | % within Scenario | 100% | 100% | 100% | 100% | 100% | 100% | 100% |

|  | Value | df | P-value |
| --- | --- | --- | --- |
| Pearson Chi-Square | 1.756 | 5 | 0.882 |

**2. Age:**

|  |  |  | Scenario | | | | | | Total |
| --- | --- | --- | --- | --- | --- | --- | --- | --- | --- |
|  |  |  | 1-1 | 1-2 | 2-1 | 2-2 | 3-1 | 3-2 |  |
| Age | Under 20 | Count | 1 | 3 | 0 | 2 | 0 | 3 | 9 |
|  |  | % within Scenario | 1.0% | 2.9% | 0.0% | 1.9% | 0.0% | 2.9% | 1.4% |
|  | 20 - 29 | Count | 32 | 28 | 29 | 41 | 37 | 22 | 189 |
|  |  | % within Scenario | 30.5% | 26.9% | 25.7% | 39.8% | 35.2% | 21.2% | 29.8% |
|  | 30 - 39 | Count | 32 | 38 | 34 | 33 | 36 | 39 | 212 |
|  |  | % within Scenario | 30.5% | 36.5% | 30.1% | 32.0% | 34.3% | 37.5% | 33.4% |
|  | 40 - 49 | Count | 16 | 16 | 28 | 14 | 20 | 21 | 115 |
|  |  | % within Scenario | 15.2% | 15.4% | 24.8% | 13.6% | 19.0% | 20.2% | 18.1% |
|  | 50 - 59 | Count | 14 | 14 | 17 | 10 | 9 | 13 | 77 |
|  |  | % within Scenario | 13.3% | 13.5% | 15.0% | 9.7% | 8.6% | 12.5% | 12.1% |
|  | 60 or older | Count | 10 | 5 | 5 | 3 | 3 | 6 | 32 |
|  |  | % within Scenario | 9.5% | 4.8% | 4.4% | 2.9% | 2.9% | 5.8% | 5.0% |
| Total | | Count | 105 | 104 | 113 | 103 | 105 | 104 | 634 |
|  |  | % within Scenario | 100% | 100% | 100% | 100% | 100% | 100% | 100% |

|  | Value | df | p-value |
| --- | --- | --- | --- |
| Pearson Chi-Square | 30.316 | 25 | 0.213 |

**3. Race/Ethnicity:**

|  |  |  | Scenario | | | | | | Total |
| --- | --- | --- | --- | --- | --- | --- | --- | --- | --- |
|  |  |  | 1-1 | 1-2 | 2-1 | 2-2 | 3-1 | 3-2 |  |
| Race/Ethnicity | White | Count | 69 | 65 | 78 | 66 | 68 | 67 | 413 |
|  |  | % within Scenario | 65.7% | 62.5% | 69.0% | 64.1% | 64.8% | 64.4% | 65.1% |
|  | African American | Count | 10 | 13 | 17 | 15 | 6 | 10 | 71 |
|  |  | % within Scenario | 9.5% | 12.5% | 15.0% | 14.6% | 5.7% | 9.6% | 11.2% |
|  | Asian | Count | 19 | 18 | 11 | 17 | 23 | 21 | 109 |
|  |  | % within Scenario | 18.1% | 17.3% | 9.7% | 16.5% | 21.9% | 20.2% | 17.2% |
|  | Hispanic | Count | 5 | 6 | 7 | 3 | 7 | 3 | 31 |
|  |  | % within Scenario | 4.8% | 5.8% | 6.2% | 2.9% | 6.7% | 2.9% | 4.9% |
|  | Mixed | Count | 2 | 1 | 0 | 2 | 0 | 2 | 7 |
|  |  | % within Scenario | 1.9% | 1.0% | 0.0% | 1.9% | 0.0% | 1.9% | 1.1% |
|  | Other | Count | 0 | 1 | 0 | 0 | 1 | 1 | 3 |
|  |  | % within Scenario | 0.0% | 1.0% | 0.0% | 0.0% | 1.0% | 1.0% | 0.5% |
| Total | | Count | 105 | 104 | 113 | 103 | 105 | 104 | 634 |
|  |  | % within Scenario | 100% | 100% | 100% | 100% | 100% | 100% | 100% |

|  | Value | df | p-value |
| --- | --- | --- | --- |
| Pearson Chi-Square | 22.366 | 25 | 0.615 |

**4. Education level:**

|  |  |  | Scenario | | | | | | Total |
| --- | --- | --- | --- | --- | --- | --- | --- | --- | --- |
|  |  |  | 1-1 | 1-2 | 2-1 | 2-2 | 3-1 | 3-2 |  |
| Highest level of education | Less than high school | Count | 0 | 1 | 0 | 0 | 1 | 0 | 2 |
|  |  | % within Scenario | 0.0% | 1.0% | 0.0% | 0.0% | 1.0% | 0.0% | 0.3% |
|  | High school graduate | Count | 9 | 6 | 9 | 4 | 3 | 8 | 39 |
|  |  | % within Scenario | 8.6% | 5.8% | 8.0% | 3.9% | 2.9% | 7.7% | 6.2% |
|  | Some college | Count | 12 | 11 | 12 | 10 | 14 | 13 | 72 |
|  |  | % within Scenario | 11.4% | 10.6% | 10.6% | 9.7% | 13.3% | 12.5% | 11.4% |
|  | 2-year degree | Count | 4 | 9 | 12 | 5 | 9 | 10 | 49 |
|  |  | % within Scenario | 3.8% | 8.7% | 10.6% | 4.9% | 8.6% | 9.6% | 7.7% |
|  | Bachelor's degree | Count | 50 | 50 | 51 | 60 | 45 | 49 | 305 |
|  |  | % within Scenario | 47.6% | 48.1% | 45.1% | 58.3% | 42.9% | 47.1% | 48.1% |
|  | Master's degree | Count | 28 | 25 | 26 | 24 | 23 | 22 | 148 |
|  |  | % within Scenario | 26.7% | 24.0% | 23.0% | 23.3% | 21.9% | 21.2% | 23.3% |
|  | Doctorate | Count | 2 | 2 | 3 | 0 | 10 | 2 | 19 |
|  |  | % within Scenario | 1.9% | 1.9% | 2.7% | 0.0% | 9.5% | 1.9% | 3.0% |
| Total | | Count | 105 | 104 | 113 | 103 | 105 | 104 | 634 |
|  |  | % within Scenario | 100% | 100% | 100% | 100% | 100% | 100% | 100% |

|  | Value | df | p-value |
| --- | --- | --- | --- |
| Pearson Chi-Square | 37.893 | 30 | 0.153 |

**5. Employment status:**

|  |  |  | Scenario | | | | | | Total |
| --- | --- | --- | --- | --- | --- | --- | --- | --- | --- |
|  |  |  | 1-1 | 1-2 | 2-1 | 2-2 | 3-1 | 3-2 |  |
| Employment | Employed full-time | Count | 74 | 71 | 82 | 79 | 73 | 79 | 458 |
|  |  | % within Scenario | 70.5% | 68.3% | 72.6% | 76.7% | 69.5% | 76.0% | 72.2% |
|  | Employed part-time | Count | 13 | 20 | 15 | 13 | 18 | 12 | 91 |
|  |  | % within Scenario | 12.4% | 19.2% | 13.3% | 12.6% | 17.1% | 11.5% | 14.4% |
|  | Unemployed | Count | 11 | 7 | 11 | 4 | 9 | 6 | 48 |
|  |  | % within Scenario | 10.5% | 6.7% | 9.7% | 3.9% | 8.6% | 5.8% | 7.6% |
|  | Retired | Count | 5 | 2 | 2 | 1 | 1 | 3 | 14 |
|  |  | % within Scenario | 4.8% | 1.9% | 1.8% | 1.0% | 1.0% | 2.9% | 2.2% |
|  | Student | Count | 2 | 4 | 3 | 6 | 4 | 4 | 23 |
|  |  | % within Scenario | 1.9% | 3.8% | 2.7% | 5.8% | 3.8% | 3.8% | 3.6% |
| Total | | Count | 105 | 104 | 113 | 103 | 105 | 104 | 634 |
|  |  | % within Scenario | 100% | 100% | 100% | 100% | 100% | 100% | 100% |

|  | Value | df | p-value |
| --- | --- | --- | --- |
| Pearson  Chi-Square | 16.202 | 20 | 0.704 |

**6. Annual household income:**

|  |  |  | Scenario | | | | | | Total |
| --- | --- | --- | --- | --- | --- | --- | --- | --- | --- |
|  |  |  | 1-1 | 1-2 | 2-1 | 2-2 | 3-1 | 3-2 |  |
| Annual household income | Less than $25,000 | Count | 17 | 18 | 14 | 11 | 20 | 16 | 96 |
|  |  | % within Scenario | 16.2% | 17.3% | 12.4% | 10.7% | 19.0% | 15.4% | 15.1% |
|  | $25,000 - $49,999 | Count | 29 | 20 | 36 | 31 | 26 | 22 | 164 |
|  |  | % within Scenario | 27.6% | 19.2% | 31.9% | 30.1% | 24.8% | 21.2% | 25.9% |
|  | $50,000 - $74,999 | Count | 19 | 28 | 20 | 30 | 20 | 24 | 141 |
|  |  | % within Scenario | 18.1% | 26.9% | 17.7% | 29.1% | 19.0% | 23.1% | 22.2% |
|  | $75,000 - $99,999 | Count | 20 | 18 | 24 | 14 | 19 | 19 | 114 |
|  |  | % within Scenario | 19.0% | 17.3% | 21.2% | 13.6% | 18.1% | 18.3% | 18.0% |
|  | $100,000 - $150,000 | Count | 15 | 17 | 14 | 11 | 13 | 17 | 87 |
|  |  | % within Scenario | 14.3% | 16.3% | 12.4% | 10.7% | 12.4% | 16.3% | 13.7% |
|  | More than $150,000 | Count | 5 | 3 | 5 | 6 | 7 | 6 | 32 |
|  |  | % within Scenario | 4.8% | 2.9% | 4.4% | 5.8% | 6.7% | 5.8% | 5.0% |
| Total | | Count | 105 | 104 | 113 | 103 | 105 | 104 | 634 |
|  |  | % within Scenario | 100% | 100% | 100% | 100% | 100% | 100% | 100% |

|  | Value | df | p-value |
| --- | --- | --- | --- |
| Pearson Chi-Square | 19.852 | 25 | 0.755 |

**7. Personal Innovativeness:**

|  | Mean | Std. Deviation | Std. Error | 95% Confidence Interval for Mean | | Minimum | Maximum |
| --- | --- | --- | --- | --- | --- | --- | --- |
|  |  |  |  | Lower Bound | Upper Bound |  |  |
| 1-1 | 13.43 | 4.181 | 0.380 | 12.68 | 14.18 | 4 | 20 |
| 1-2 | 13.28 | 4.034 | 0.367 | 12.55 | 14.01 | 4 | 20 |
| 2-1 | 14.50 | 3.207 | 0.292 | 13.92 | 15.07 | 5 | 20 |
| 2-2 | 13.89 | 3.566 | 0.324 | 13.25 | 14.53 | 4 | 20 |
| 3-1 | 13.98 | 3.511 | 0.319 | 13.34 | 14.61 | 6 | 20 |
| 3-2 | 14.01 | 3.639 | 0.331 | 13.35 | 14.66 | 6 | 20 |
| Total | 13.85 | 3.713 | 0.138 | 13.58 | 14.12 | 4 | 20 |

|  | Sum of Squares | df | Mean Square | F | Sig. |
| --- | --- | --- | --- | --- | --- |
| Between Groups | 116.161 | 5 | 23.232 | 1.477 | 0.195 |
| Within Groups | 9877.868 | 628 | 15.729 |  |  |
| Total | 9994.029 | 633 |  |  |  |
