# Supplementary material for: Patients’ Perceptions Toward Human–Artificial Intelligence Interaction in Health Care: Experimental Study
Source: J Med Internet Res. 2021 Nov 25;23(11):e25856. doi: 10.2196/25856 (PMC8663518; doi:10.2196/25856)
Supplement: Multimedia Appendix 3 [file jmir_v23i11e25856_app3.docx]

**Appendix 3:**

**Detailed Scheffe posthoc test results:**

| **Multiple Comparisons** | | | | | | | |
| --- | --- | --- | --- | --- | --- | --- | --- |
| Scheffe |  |  |  |  |  |  |  |
| Dependent Variable | (I) Scenario | (J) Scenario | Mean Difference (I-J) | Std. Error | Sig. | 95% Confidence Interval | |
|  |  |  |  |  |  | Lower Bound | Upper Bound |
| Perceived Privacy Concerns | 1-1 | 1-2 | -1.529 | 0.873 | 0.690 | -4.44 | 1.39 |
|  |  | 2-1 | -1.752 | 0.856 | 0.523 | -4.61 | 1.10 |
|  |  | 2-2 | -0.816 | 0.876 | 0.972 | -3.74 | 2.11 |
|  |  | 3-1 | 1.314 | 0.871 | 0.810 | -1.59 | 4.22 |
|  |  | 3-2 | -0.471 | 0.873 | 0.998 | -3.39 | 2.44 |
|  | 1-2 | 1-1 | 1.529 | 0.873 | 0.690 | -1.39 | 4.44 |
|  |  | 2-1 | -0.223 | 0.858 | 1.000 | -3.09 | 2.64 |
|  |  | 2-2 | 0.713 | 0.878 | 0.985 | -2.22 | 3.64 |
|  |  | 3-1 | 2.843 | 0.873 | 0.061 | -0.07 | 5.76 |
|  |  | 3-2 | 1.058 | 0.875 | 0.917 | -1.86 | 3.98 |
|  | 2-1 | 1-1 | 1.752 | 0.856 | 0.523 | -1.10 | 4.61 |
|  |  | 1-2 | 0.223 | 0.858 | 1.000 | -2.64 | 3.09 |
|  |  | 2-2 | 0.936 | 0.860 | 0.946 | -1.93 | 3.81 |
|  |  | 3-1 | 3.066^*^ | 0.856 | 0.026 | 0.21 | 5.92 |
|  |  | 3-2 | 1.280 | 0.858 | 0.817 | -1.58 | 4.14 |
|  | 2-2 | 1-1 | 0.816 | 0.876 | 0.972 | -2.11 | 3.74 |
|  |  | 1-2 | -0.713 | 0.878 | 0.985 | -3.64 | 2.22 |
|  |  | 2-1 | -0.936 | 0.860 | 0.946 | -3.81 | 1.93 |
|  |  | 3-1 | 2.130 | 0.876 | 0.316 | -0.79 | 5.05 |
|  |  | 3-2 | 0.344 | 0.878 | 1.000 | -2.58 | 3.27 |
|  | 3-1 | 1-1 | -1.314 | 0.871 | 0.810 | -4.22 | 1.59 |
|  |  | 1-2 | -2.843 | 0.873 | 0.061 | -5.76 | 0.07 |
|  |  | 2-1 | -3.066^*^ | 0.856 | 0.026 | -5.92 | -0.21 |
|  |  | 2-2 | -2.130 | 0.876 | 0.316 | -5.05 | 0.79 |
|  |  | 3-2 | -1.786 | 0.873 | 0.524 | -4.70 | 1.13 |
|  | 3-2 | 1-1 | 0.471 | 0.873 | 0.998 | -2.44 | 3.39 |
|  |  | 1-2 | -1.058 | 0.875 | 0.917 | -3.98 | 1.86 |
|  |  | 2-1 | -1.280 | 0.858 | 0.817 | -4.14 | 1.58 |
|  |  | 2-2 | -0.344 | 0.878 | 1.000 | -3.27 | 2.58 |
|  |  | 3-1 | 1.786 | 0.873 | 0.524 | -1.13 | 4.70 |
| Perceived Trust | 1-1 | 1-2 | 1.063 | 0.661 | 0.763 | -1.14 | 3.27 |
|  |  | 2-1 | -0.789 | 0.647 | 0.915 | -2.95 | 1.37 |
|  |  | 2-2 | -0.340 | 0.662 | 0.998 | -2.55 | 1.87 |
|  |  | 3-1 | -2.152 | 0.659 | 0.060 | -4.35 | 0.05 |
|  |  | 3-2 | -1.707 | 0.661 | 0.248 | -3.91 | 0.50 |
|  | 1-2 | 1-1 | -1.063 | 0.661 | 0.763 | -3.27 | 1.14 |
|  |  | 2-1 | -1.851 | 0.649 | 0.151 | -4.02 | 0.32 |
|  |  | 2-2 | -1.402 | 0.664 | 0.486 | -3.62 | 0.81 |
|  |  | 3-1 | -3.215^*^ | 0.661 | 0.000 | -5.42 | -1.01 |
|  |  | 3-2 | -2.769^*^ | 0.662 | 0.004 | -4.98 | -0.56 |
|  | 2-1 | 1-1 | 0.789 | 0.647 | 0.915 | -1.37 | 2.95 |
|  |  | 1-2 | 1.851 | 0.649 | 0.151 | -0.32 | 4.02 |
|  |  | 2-2 | 0.449 | 0.651 | 0.993 | -1.72 | 2.62 |
|  |  | 3-1 | -1.364 | 0.647 | 0.489 | -3.52 | 0.80 |
|  |  | 3-2 | -0.918 | 0.649 | 0.849 | -3.08 | 1.25 |
|  | 2-2 | 1-1 | 0.340 | 0.662 | 0.998 | -1.87 | 2.55 |
|  |  | 1-2 | 1.402 | 0.664 | 0.486 | -0.81 | 3.62 |
|  |  | 2-1 | -0.449 | 0.651 | 0.993 | -2.62 | 1.72 |
|  |  | 3-1 | -1.813 | 0.662 | 0.188 | -4.02 | 0.40 |
|  |  | 3-2 | -1.367 | 0.664 | 0.516 | -3.58 | 0.85 |
|  | 3-1 | 1-1 | 2.152 | 0.659 | 0.060 | -0.05 | 4.35 |
|  |  | 1-2 | 3.215^*^ | 0.661 | 0.000 | 1.01 | 5.42 |
|  |  | 2-1 | 1.364 | 0.647 | 0.489 | -0.80 | 3.52 |
|  |  | 2-2 | 1.813 | 0.662 | 0.188 | -0.40 | 4.02 |
|  |  | 3-2 | 0.446 | 0.661 | 0.994 | -1.76 | 2.65 |
|  | 3-2 | 1-1 | 1.707 | 0.661 | 0.248 | -0.50 | 3.91 |
|  |  | 1-2 | 2.769^*^ | 0.662 | 0.004 | 0.56 | 4.98 |
|  |  | 2-1 | 0.918 | 0.649 | 0.849 | -1.25 | 3.08 |
|  |  | 2-2 | 1.367 | 0.664 | 0.516 | -0.85 | 3.58 |
|  |  | 3-1 | -0.446 | 0.661 | 0.994 | -2.65 | 1.76 |
| Percevied Communication Barriers | 1-1 | 1-2 | 0.342 | 0.718 | 0.999 | -2.05 | 2.74 |
|  |  | 2-1 | -0.625 | 0.704 | 0.978 | -2.97 | 1.72 |
|  |  | 2-2 | -0.115 | 0.720 | 1.000 | -2.52 | 2.29 |
|  |  | 3-1 | 2.752^*^ | 0.716 | 0.012 | 0.36 | 5.14 |
|  |  | 3-2 | 2.861^*^ | 0.718 | 0.008 | 0.46 | 5.26 |
|  | 1-2 | 1-1 | -0.342 | 0.718 | 0.999 | -2.74 | 2.05 |
|  |  | 2-1 | -0.967 | 0.705 | 0.866 | -3.32 | 1.39 |
|  |  | 2-2 | -0.457 | 0.722 | 0.995 | -2.87 | 1.95 |
|  |  | 3-1 | 2.410^*^ | 0.718 | 0.048 | 0.01 | 4.81 |
|  |  | 3-2 | 2.519^*^ | 0.720 | 0.033 | 0.12 | 4.92 |
|  | 2-1 | 1-1 | 0.625 | 0.704 | 0.978 | -1.72 | 2.97 |
|  |  | 1-2 | 0.967 | 0.705 | 0.866 | -1.39 | 3.32 |
|  |  | 2-2 | 0.509 | 0.707 | 0.991 | -1.85 | 2.87 |
|  |  | 3-1 | 3.377^*^ | 0.704 | 0.000 | 1.03 | 5.73 |
|  |  | 3-2 | 3.486^*^ | 0.705 | 0.000 | 1.13 | 5.84 |
|  | 2-2 | 1-1 | 0.115 | 0.720 | 1.000 | -2.29 | 2.52 |
|  |  | 1-2 | 0.457 | 0.722 | 0.995 | -1.95 | 2.87 |
|  |  | 2-1 | -0.509 | 0.707 | 0.991 | -2.87 | 1.85 |
|  |  | 3-1 | 2.867^*^ | 0.720 | 0.008 | 0.46 | 5.27 |
|  |  | 3-2 | 2.976^*^ | 0.722 | 0.005 | 0.57 | 5.38 |
|  | 3-1 | 1-1 | -2.752^*^ | 0.716 | 0.012 | -5.14 | -0.36 |
|  |  | 1-2 | -2.410^*^ | 0.718 | 0.048 | -4.81 | -0.01 |
|  |  | 2-1 | -3.377^*^ | 0.704 | 0.000 | -5.73 | -1.03 |
|  |  | 2-2 | -2.867^*^ | 0.720 | 0.008 | -5.27 | -0.46 |
|  |  | 3-2 | 0.109 | 0.718 | 1.000 | -2.29 | 2.51 |
|  | 3-2 | 1-1 | -2.861^*^ | 0.718 | 0.008 | -5.26 | -0.46 |
|  |  | 1-2 | -2.519^*^ | 0.720 | 0.033 | -4.92 | -0.12 |
|  |  | 2-1 | -3.486^*^ | 0.705 | 0.000 | -5.84 | -1.13 |
|  |  | 2-2 | -2.976^*^ | 0.722 | 0.005 | -5.38 | -0.57 |
|  |  | 3-1 | -0.109 | 0.718 | 1.000 | -2.51 | 2.29 |
| Perceived Transparency of Regulatory Standards | 1-1 | 1-2 | 0.270 | 0.698 | 1.000 | -2.06 | 2.60 |
|  |  | 2-1 | -0.184 | 0.684 | 1.000 | -2.47 | 2.10 |
|  |  | 2-2 | 0.332 | 0.700 | 0.999 | -2.00 | 2.67 |
|  |  | 3-1 | 3.038^*^ | 0.696 | 0.002 | 0.71 | 5.36 |
|  |  | 3-2 | 2.905^*^ | 0.698 | 0.004 | 0.57 | 5.24 |
|  | 1-2 | 1-1 | -0.270 | 0.698 | 1.000 | -2.60 | 2.06 |
|  |  | 2-1 | -0.454 | 0.686 | 0.994 | -2.74 | 1.84 |
|  |  | 2-2 | 0.062 | 0.702 | 1.000 | -2.28 | 2.40 |
|  |  | 3-1 | 2.768^*^ | 0.698 | 0.008 | 0.44 | 5.10 |
|  |  | 3-2 | 2.635^*^ | 0.700 | 0.015 | 0.30 | 4.97 |
|  | 2-1 | 1-1 | 0.184 | 0.684 | 1.000 | -2.10 | 2.47 |
|  |  | 1-2 | 0.454 | 0.686 | 0.994 | -1.84 | 2.74 |
|  |  | 2-2 | 0.516 | 0.687 | 0.990 | -1.78 | 2.81 |
|  |  | 3-1 | 3.222^*^ | 0.684 | 0.001 | 0.94 | 5.51 |
|  |  | 3-2 | 3.088^*^ | 0.686 | 0.001 | 0.80 | 5.38 |
|  | 2-2 | 1-1 | -0.332 | 0.700 | 0.999 | -2.67 | 2.00 |
|  |  | 1-2 | -0.062 | 0.702 | 1.000 | -2.40 | 2.28 |
|  |  | 2-1 | -0.516 | 0.687 | 0.990 | -2.81 | 1.78 |
|  |  | 3-1 | 2.706^*^ | 0.700 | 0.011 | 0.37 | 5.04 |
|  |  | 3-2 | 2.573^*^ | 0.702 | 0.020 | 0.23 | 4.91 |
|  | 3-1 | 1-1 | -3.038^*^ | 0.696 | 0.002 | -5.36 | -0.71 |
|  |  | 1-2 | -2.768^*^ | 0.698 | 0.008 | -5.10 | -0.44 |
|  |  | 2-1 | -3.222^*^ | 0.684 | 0.001 | -5.51 | -0.94 |
|  |  | 2-2 | -2.706^*^ | 0.700 | 0.011 | -5.04 | -0.37 |
|  |  | 3-2 | -0.133 | 0.698 | 1.000 | -2.46 | 2.20 |
|  | 3-2 | 1-1 | -2.905^*^ | 0.698 | 0.004 | -5.24 | -0.57 |
|  |  | 1-2 | -2.635^*^ | 0.700 | 0.015 | -4.97 | -0.30 |
|  |  | 2-1 | -3.088^*^ | 0.686 | 0.001 | -5.38 | -0.80 |
|  |  | 2-2 | -2.573^*^ | 0.702 | 0.020 | -4.91 | -0.23 |
|  |  | 3-1 | 0.133 | 0.698 | 1.000 | -2.20 | 2.46 |
| Perceived Liability Issues | 1-1 | 1-2 | 0.507 | 0.848 | 0.996 | -2.33 | 3.34 |
|  |  | 2-1 | -0.805 | 0.831 | 0.967 | -3.58 | 1.97 |
|  |  | 2-2 | 0.684 | 0.851 | 0.986 | -2.15 | 3.52 |
|  |  | 3-1 | 2.724 | 0.846 | 0.067 | -0.10 | 5.55 |
|  |  | 3-2 | 2.921^*^ | 0.848 | 0.038 | 0.09 | 5.75 |
|  | 1-2 | 1-1 | -0.507 | 0.848 | 0.996 | -3.34 | 2.33 |
|  |  | 2-1 | -1.312 | 0.833 | 0.780 | -4.09 | 1.47 |
|  |  | 2-2 | 0.177 | 0.853 | 1.000 | -2.67 | 3.02 |
|  |  | 3-1 | 2.217 | 0.848 | 0.236 | -0.62 | 5.05 |
|  |  | 3-2 | 2.413 | 0.851 | 0.155 | -0.43 | 5.25 |
|  | 2-1 | 1-1 | 0.805 | 0.831 | 0.967 | -1.97 | 3.58 |
|  |  | 1-2 | 1.312 | 0.833 | 0.780 | -1.47 | 4.09 |
|  |  | 2-2 | 1.489 | 0.836 | 0.673 | -1.30 | 4.28 |
|  |  | 3-1 | 3.529^*^ | 0.831 | 0.003 | 0.75 | 6.30 |
|  |  | 3-2 | 3.725^*^ | 0.833 | 0.001 | 0.94 | 6.51 |
|  | 2-2 | 1-1 | -0.684 | 0.851 | 0.986 | -3.52 | 2.15 |
|  |  | 1-2 | -0.177 | 0.853 | 1.000 | -3.02 | 2.67 |
|  |  | 2-1 | -1.489 | 0.836 | 0.673 | -4.28 | 1.30 |
|  |  | 3-1 | 2.039 | 0.851 | 0.333 | -0.80 | 4.88 |
|  |  | 3-2 | 2.236 | 0.853 | 0.231 | -0.61 | 5.08 |
|  | 3-1 | 1-1 | -2.724 | 0.846 | 0.067 | -5.55 | 0.10 |
|  |  | 1-2 | -2.217 | 0.848 | 0.236 | -5.05 | 0.62 |
|  |  | 2-1 | -3.529^*^ | 0.831 | 0.003 | -6.30 | -0.75 |
|  |  | 2-2 | -2.039 | 0.851 | 0.333 | -4.88 | 0.80 |
|  |  | 3-2 | 0.197 | 0.848 | 1.000 | -2.64 | 3.03 |
|  | 3-2 | 1-1 | -2.921^*^ | 0.848 | 0.038 | -5.75 | -0.09 |
|  |  | 1-2 | -2.413 | 0.851 | 0.155 | -5.25 | 0.43 |
|  |  | 2-1 | -3.725^*^ | 0.833 | 0.001 | -6.51 | -0.94 |
|  |  | 2-2 | -2.236 | 0.853 | 0.231 | -5.08 | 0.61 |
|  |  | 3-1 | -0.197 | 0.848 | 1.000 | -3.03 | 2.64 |
| Perceived Benefits | 1-1 | 1-2 | 0.832 | 0.851 | 0.966 | -2.01 | 3.67 |
|  |  | 2-1 | -1.445 | 0.834 | 0.700 | -4.23 | 1.34 |
|  |  | 2-2 | 0.292 | 0.853 | 1.000 | -2.56 | 3.14 |
|  |  | 3-1 | -1.629 | 0.849 | 0.597 | -4.46 | 1.21 |
|  |  | 3-2 | -1.562 | 0.851 | 0.643 | -4.40 | 1.28 |
|  | 1-2 | 1-1 | -0.832 | 0.851 | 0.966 | -3.67 | 2.01 |
|  |  | 2-1 | -2.277 | 0.836 | 0.193 | -5.07 | 0.51 |
|  |  | 2-2 | -0.540 | 0.855 | 0.995 | -3.39 | 2.31 |
|  |  | 3-1 | -2.461 | 0.851 | 0.139 | -5.30 | 0.38 |
|  |  | 3-2 | -2.394 | 0.853 | 0.165 | -5.24 | 0.45 |
|  | 2-1 | 1-1 | 1.445 | 0.834 | 0.700 | -1.34 | 4.23 |
|  |  | 1-2 | 2.277 | 0.836 | 0.193 | -0.51 | 5.07 |
|  |  | 2-2 | 1.737 | 0.838 | 0.508 | -1.06 | 4.53 |
|  |  | 3-1 | -0.184 | 0.834 | 1.000 | -2.97 | 2.60 |
|  |  | 3-2 | -0.117 | 0.836 | 1.000 | -2.91 | 2.67 |
|  | 2-2 | 1-1 | -0.292 | 0.853 | 1.000 | -3.14 | 2.56 |
|  |  | 1-2 | 0.540 | 0.855 | 0.995 | -2.31 | 3.39 |
|  |  | 2-1 | -1.737 | 0.838 | 0.508 | -4.53 | 1.06 |
|  |  | 3-1 | -1.920 | 0.853 | 0.409 | -4.77 | 0.93 |
|  |  | 3-2 | -1.854 | 0.855 | 0.454 | -4.71 | 1.00 |
|  | 3-1 | 1-1 | 1.629 | 0.849 | 0.597 | -1.21 | 4.46 |
|  |  | 1-2 | 2.461 | 0.851 | 0.139 | -0.38 | 5.30 |
|  |  | 2-1 | 0.184 | 0.834 | 1.000 | -2.60 | 2.97 |
|  |  | 2-2 | 1.920 | 0.853 | 0.409 | -0.93 | 4.77 |
|  |  | 3-2 | 0.066 | 0.851 | 1.000 | -2.77 | 2.91 |
|  | 3-2 | 1-1 | 1.562 | 0.851 | 0.643 | -1.28 | 4.40 |
|  |  | 1-2 | 2.394 | 0.853 | 0.165 | -0.45 | 5.24 |
|  |  | 2-1 | 0.117 | 0.836 | 1.000 | -2.67 | 2.91 |
|  |  | 2-2 | 1.854 | 0.855 | 0.454 | -1.00 | 4.71 |
|  |  | 3-1 | -0.066 | 0.851 | 1.000 | -2.91 | 2.77 |
| Intention to Use | 1-1 | 1-2 | 0.764 | 0.680 | 0.938 | -1.50 | 3.03 |
|  |  | 2-1 | -0.793 | 0.666 | 0.922 | -3.02 | 1.43 |
|  |  | 2-2 | 0.038 | 0.681 | 1.000 | -2.24 | 2.31 |
|  |  | 3-1 | -2.752^*^ | 0.678 | 0.006 | -5.02 | -0.49 |
|  |  | 3-2 | -2.726^*^ | 0.680 | 0.007 | -5.00 | -0.46 |
|  | 1-2 | 1-1 | -0.764 | 0.680 | 0.938 | -3.03 | 1.50 |
|  |  | 2-1 | -1.557 | 0.668 | 0.366 | -3.79 | 0.67 |
|  |  | 2-2 | -0.726 | 0.683 | 0.951 | -3.01 | 1.55 |
|  |  | 3-1 | -3.516^*^ | 0.680 | 0.000 | -5.79 | -1.25 |
|  |  | 3-2 | -3.490^*^ | 0.681 | 0.000 | -5.76 | -1.22 |
|  | 2-1 | 1-1 | 0.793 | 0.666 | 0.922 | -1.43 | 3.02 |
|  |  | 1-2 | 1.557 | 0.668 | 0.366 | -0.67 | 3.79 |
|  |  | 2-2 | 0.831 | 0.669 | 0.908 | -1.40 | 3.06 |
|  |  | 3-1 | -1.960 | 0.666 | 0.125 | -4.18 | 0.26 |
|  |  | 3-2 | -1.934 | 0.668 | 0.138 | -4.16 | 0.29 |
|  | 2-2 | 1-1 | -0.038 | 0.681 | 1.000 | -2.31 | 2.24 |
|  |  | 1-2 | 0.726 | 0.683 | 0.951 | -1.55 | 3.01 |
|  |  | 2-1 | -0.831 | 0.669 | 0.908 | -3.06 | 1.40 |
|  |  | 3-1 | -2.790^*^ | 0.681 | 0.005 | -5.06 | -0.52 |
|  |  | 3-2 | -2.764^*^ | 0.683 | 0.006 | -5.04 | -0.48 |
|  | 3-1 | 1-1 | 2.752^*^ | 0.678 | 0.006 | 0.49 | 5.02 |
|  |  | 1-2 | 3.516^*^ | 0.680 | 0.000 | 1.25 | 5.79 |
|  |  | 2-1 | 1.960 | 0.666 | 0.125 | -0.26 | 4.18 |
|  |  | 2-2 | 2.790^*^ | 0.681 | 0.005 | 0.52 | 5.06 |
|  |  | 3-2 | 0.026 | 0.680 | 1.000 | -2.24 | 2.29 |
|  | 3-2 | 1-1 | 2.726^*^ | 0.680 | 0.007 | 0.46 | 5.00 |
|  |  | 1-2 | 3.490^*^ | 0.681 | 0.000 | 1.22 | 5.76 |
|  |  | 2-1 | 1.934 | 0.668 | 0.138 | -0.29 | 4.16 |
|  |  | 2-2 | 2.764^*^ | 0.683 | 0.006 | 0.48 | 5.04 |
|  |  | 3-1 | -0.026 | 0.680 | 1.000 | -2.29 | 2.24 |

**Outcome variable differences across proposed scenarios: Bar charts**
